# Whole Body Motor Adaptation in Goldfish Using Fish Operated Vehicle

**DOI:** 10.1111/ejn.70241

**Published:** 2025-09-02

**Authors:** Zhuoxin Liu, Shachar Givon, Ronen Segev, Opher Donchin

**Affiliations:** ^1^ Department of Mechatronics Engineering Ben‐Gurion University of the Negev Be'er Sheva Israel; ^2^ Department of Biomedical Engineering Ben‐Gurion University of the Negev Be'er Sheva Israel; ^3^ School of Brain Sciences and Cognition Ben‐Gurion University of the Negev Be'er Sheva Israel; ^4^ Department of Life Sciences Ben‐Gurion University of the Negev Be'er Sheva Israel

**Keywords:** animal learning, fish, motor behavior, motor learning

## Abstract

Motor adaptation is crucial for animals to move in diverse environments, including fish. Here, we develop a novel experimental platform that allows for precise control of sensorimotor transformations and direct comparison with established paradigms used in mammalian studies. We show that goldfish operating a fish operated vehicle (FOV) adapt swimming behavior to achieve targets when vehicle movement is perturbed by a rotational transformation. Goldfish gradually adjusted their swimming patterns to compensate for the perturbation and had aftereffects when the perturbation was removed. Fish showed improved performance when the perturbation was reintroduced, although their initial learning rate in the second exposure was slower compared to the first exposure. These findings reveal that although goldfish can adapt to novel dynamics, their adaptation mechanisms may differ from those of mammals. This study broadens our understanding of motor adaptation across species, contributing to a more comprehensive view of motor learning in vertebrates.

## Graphical abstract

A fish operated vehicle (FOV) is a mobile platform that enables fish to navigate by translating their swimming movements into vehicle motion. Goldfish operating a FOV adapt swimming behavior to achieve targets when vehicle movement is perturbed by a rotational transformation. Goldfish gradually adjusted their swimming patterns to compensate for the perturbation and had aftereffects when the perturbation is removed. Fish showed improved performance when the perturbation was reintroduced, although their initial learning rate in the second exposure was slower compared to the first exposure. These findings reveal that although goldfish can adapt to novel dynamics, their adaptation mechanisms may differ from those of mammals.

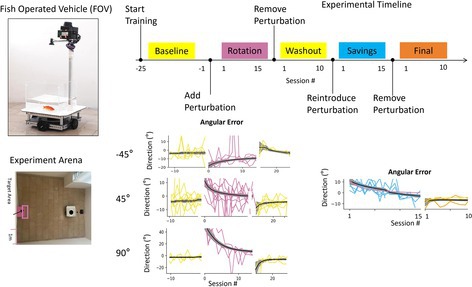

**Abbreviations**
EAL: estimated additional length
eff: efficiency of the session
ET: the distance from end point to target point
FOV: fish operated vehicle
HDI: highest density interval
MCMC: Markov Chain Monte Carlo
ROPE: region of practical equivalence
SE: the distance from start point to end point

## 1 Introduction

Animals routinely adapt to novel sensorimotor feedback. For example, humans can adapt to walking on split‐belt treadmills (Choi and Bastian 2007; Finley et al. 2013; Reisman et al. 2007), reaching for targets under artificially induced force‐fields (Donchin et al. 2003; Schween et al. 2020; Spampinato and Celnik 2021) or altered vestibular feedback (Blouin et al. 2015; Smith and Reynolds 2017; Welgampola and Colebatch 2001). Adaptation has also been shown in many other species (Grabowska et al. 2012; Herzfeld et al. 2018; Salem et al. 2022).

In aquatic environments, fish subjected to consistent flows require an ability to maintain stability. This stability can be achieved through self‐correcting mechanisms or actively with powered movements (Jindrich and Full 2002; Webb 2004). Prior studies have revealed mechanisms and strategies of fish learning to compensate for these effects in locomotion or eye movements (Aksay et al. 2007; Fernald 1988; McHenry and Liao 2014). In a recent study, Yang et al. (2024) examined how weakly electric fish changed their refuge tracking behavior in response to an innovative experimental paradigm. The results imply that weakly electric fish adaptively retune their control system under augmented feedback for maintaining robust task‐level control performance. Thus, it seems fish can adapt to a variety of their behaviors in the face of a changing environment.

Here, we extend this research in several important directions. First, we use a well‐studied fish for which much anatomical information and many genetic tools are available (Blanco et al. 2018; Broglio et al. 2010; Salas et al. 2006) but for which adaptation has not yet been studied. Second, we study a paradigm in which the fish is controlling a vehicle (FOV), behavior that is not natural for a fish and for which there is no strong reason to expect hardwired adaptation mechanisms. Third, we explore whether fish adaptation shows savings, one of the hallmarks of adaptation in other animals (Albert et al. 2022).

The FOV is a self‐propelled platform whose motion is controlled by the fish placed in an onboard water tank; that is, it is basically a car that allows a fish to be mobile in a terrestrial environment. Control is mediated by an onboard camera and a computer vision system that detects the fish's position in real time and activates the vehicle motors accordingly. Whenever the fish is near one of the water tank walls and facing outwards, the FOV is automatically propelled in this direction. When this mapping is learned by the fish, it can drive the vehicle in a terrestrial environment.

The FOV provides a unique opportunity to study motor adaptation in fish, as its computerized control system can be modified to include constant distortions in the mapping between fish behavior and vehicle response (Crawford et al. 2020; Givon et al. 2022). This approach allows us to establish a motor adaptation paradigm that is comparatively reliable and comprehensive.

There are several basic properties of motor adaptation that we expect to see if motor adaptation in the FOV is equivalent to the widely established mechanisms of motor adaptation in other systems. Washout refers to the gradual decay or elimination of motor adaptation when the perturbation is removed. In addition, saving (also called “savings”) is the phenomenon where relearning occurs better and faster upon reexposure to a previously encountered perturbation (Maresch et al. 2020). Our study aims to address the following questions: Can goldfish learn from error while operating the vehicle with constant perturbation? Would fish in the FOV show an after‐effect in a washout session? Would fish relearn faster after the first adaptation? Can we observe persistent effects on fish swimming after the first adaptation? As we show, the answers to these questions are yes, yes, no, and yes essentially—establishing that goldfish adapt to perturbations in FOV control but raising the possibility that they do so with mechanisms that are different from classical adaptation in mammals. Our research contributes to the growing body of knowledge on fish motor adaptation and locomotion in altered environments, offering insights into the adaptive capabilities of aquatic organisms in novel situations. Furthermore, this study may help bridge the gap between our understanding of motor adaptation in fish and in higher vertebrates, potentially revealing evolutionary aspects of motor learning and control.

## 2 Materials and Methods

### 2.1 Animals

All the experiments were approved by the Ben‐Gurion University of the Negev Institutional Animal Care and Use Committee and were in accordance with the government regulations of the State of Israel. A total of 12 goldfish ( *Carassius auratus* ), 15–18 cm in body length, and 80–120 g body weight were used in the study. The fish were housed in pairs in water tanks kept at room temperature with an artificial 12/12 h day‐night light cycle. The fish were relocated to the FOV water tank for experiments. For 2 weeks before beginning experiments, each fish was habituated to the food used as a reward.

### 2.2 Fish Operated Vehicle

The fish operated vehicle (FOV) is a custom‐built apparatus designed to allow a fish to control its movement within a defined arena. The FOV consists of a robust chassis measuring 40 × 40 × 19 cm, which supports a Perspex water tank (35 × 35 × 28 cm) positioned on a central platform. The tank is filled with water to a depth of 15 cm, a level chosen specifically to minimize surface waves caused by the vehicle's movement.

**FIGURE 1:**
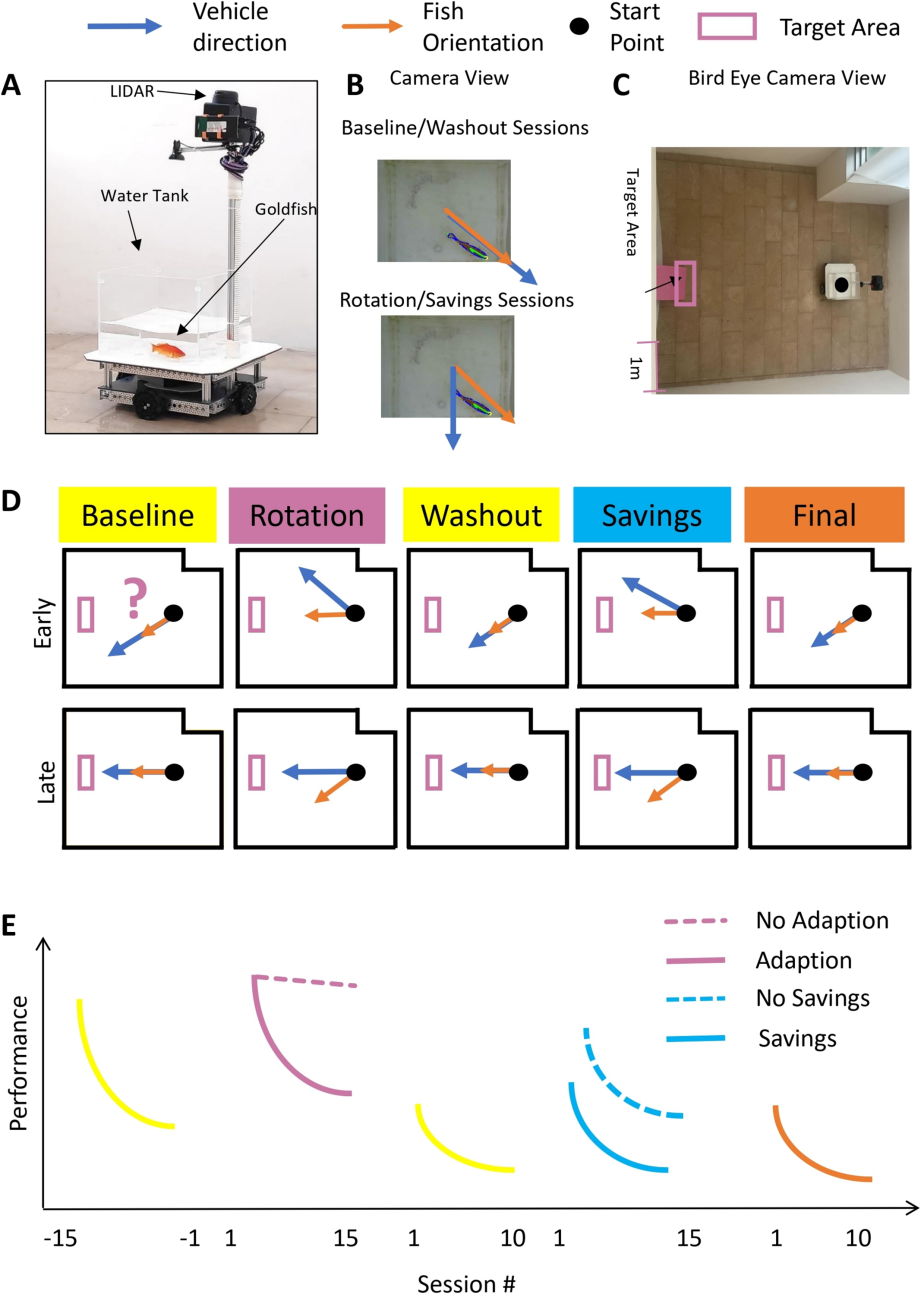
Experimental setup and working hypothesis. (A) The fish operated vehicle is composed of a chassis with four electric motors equipped with omni wheels and a camera together with a LIDAR to collect data on fish relative position within the water tank and vehicle relative position in the arena. (B) View of a fish from the camera: fish orientation (orange) and FOV direction (blue). The FOV direction is controlled by the orientation of the fish. In the baseline/washout/final sessions, when the fish swims near a wall of the tank facing outward, the vehicle moves in the corresponding direction. In the rotation/savings sessions, the control system was adjusted to rotate the vehicle's movement direction 45° to the right relative to the direction the fish is facing. (C) The FOV and arena, bird's eye view. (D) The experiment includes five stages: baseline, rotation, washout, savings, and final. Each stage had corresponding sessions. Each session had six trials. The question mark in the early stage of Baseline represents the of behavior in a naive fish. The plots reflect the expected behavior at the beginning and end of each stage according to our working hypotheses. (E) The working hypothesis of the FOV experiment. If the fish are not able to adapt to the rotated perturbation, the performance will be like the dash reddish purple line. If the fish has savings, the performance will be like the solid sky blue line.

Four Brushed DC motors are mounted beneath the platform, each connected to one of four omni wheels (4″ OMNI, 595671, Actobotics) positioned on the sides of the chassis, enabling precise, omnidirectional movement (Figure 1A). Above the tank, a Raspberry Pi 3B+ computer, a LIDAR sensor (RPLIDAR A2M8, Slamtec), and a power bank (10 Ah Type‐C 18W PD) are secured to a side pole extending 40 cm above the platform. A camera (C270 HD WEBCAM, Logitech) is mounted 20 cm inward from the pole, facing downward to capture the fish's position within the tank.

The core of the FOV's functionality lies in its computer vision system, which allows the fish to control the vehicle's movement. This system processes real‐time video from the downward‐facing camera, utilizing image segmentation and object detection to track the fish's position and orientation. When the fish swims near a wall of the tank facing outward, the vehicle moves in the corresponding direction (Figure 1B). Conversely, the vehicle remains stationary when the fish faces inward. The Raspberry Pi continuously records both the fish's position within the tank and the vehicle's position within the room for subsequent analysis.

During specific experimental sessions involving rotation, the control system was adjusted to rotate the vehicle's movement direction 45° to the right relative to the direction the fish is facing. This modification was essential for studying the fish's response to altered movement cues.

For precise localization within the experimental arena, the LIDAR sensor mounted on the FOV records the vehicle's position at a frequency of 11.3 Hz (618 rpm) with an accuracy of less than 0.5 mm. For comprehensive data analysis, bird's eye view footage of the entire experiment is also recorded using a separate camera system placed approximately 4 m above the workspace. This camera captures video at 40 frames per second with a resolution of 1920 × 1342 pixels. These recordings are downsampled to 30 fps and resized to 720 × 480 pixels during processing, serving to validate the reliability and integrity of the data collected by the FOV. The camera attached to the water tank captures high‐resolution video (30 fps, 320 × 240) of the fish's position and pose within the water tank. This information is integrated with the LIDAR data in a control loop, which updates in real‐time at a rate of 12 fps to adjust the vehicle's movement based on the fish's behavior.

Detailed information on the vehicle's motor control system, response characteristics, and the behavioral arena can be found in a previous publication (Givon et al. 2022).

### 2.3 Trial Structure

Each trial begins with uncovering a bucket that covers the FOV's water tank between trials when the FOV is at the starting point (Figure 1C). Control of the FOV is then transferred to the fish. When the fish successfully reaches the target, defined as the moment the vehicle contacts the pink board, the experimenter dispenses one food pellet into the water tank. After the fish consumes the pellet, the bucket is used to obscure visual feedback. The FOV is then manually returned to the starting point for the next trial. The maximum time given for the fish to bring the FOV to the target location is 3 min. A trial ends when any of the following conditions are met: Test time exceeds 3 min, the FOV reaches the test site boundaries, or the FOV successfully arrives at the target area. The fish is rewarded with food only upon successfully reaching the target.

### 2.4 Timeline of Experiment

Fish participated in experimental sessions on alternate days. In each experimental session, the fish had six trials. The sessions were grouped into five distinct stages: baseline, rotation, washout, savings, and final.

Individual fish exhibited significant variability in their ability to associate the food reward with the target during the baseline stages. To account for this, we established a maximum of 25 baseline sessions for fish to reach a criterion of performance. The criteria for advancing from the baseline to the rotation stage required the fish to maintain good performance standards (described in the data preprocessing section) for three consecutive sessions. Thus, the number of baseline sessions varied between fish, as each fish proceeded to the next stage once it reached the performance criteria. Of the 12 fish that began the 45° experiment, 8 completed the baseline (of the remaining fish, 3 died during baseline, and 1 failed to achieve baseline performance criteria). Of the four fish that began 90° experiment, three completed the baseline (one failed to achieve baseline performance criteria). All four fish that began −45° experiment completed the baseline. The −45° and 90° groups stopped at the washout stage. The difference between each group is the rotational perturbation size.

After the baseline, the 45° group experiments proceeded as follows:

1. Rotation stage: 15 sessions, in which FOV movement direction was rotated 45° relative to the direction commanded by the fish pose.
2. Washout stage: 10 sessions, in which FOV movement was again the direction commanded by the fish pose.
3. Savings stage: 15 sessions as in 1.
4. Final stage: 10 sessions as in 2.

### 2.5 Data Preprocessing

**FIGURE 2:**
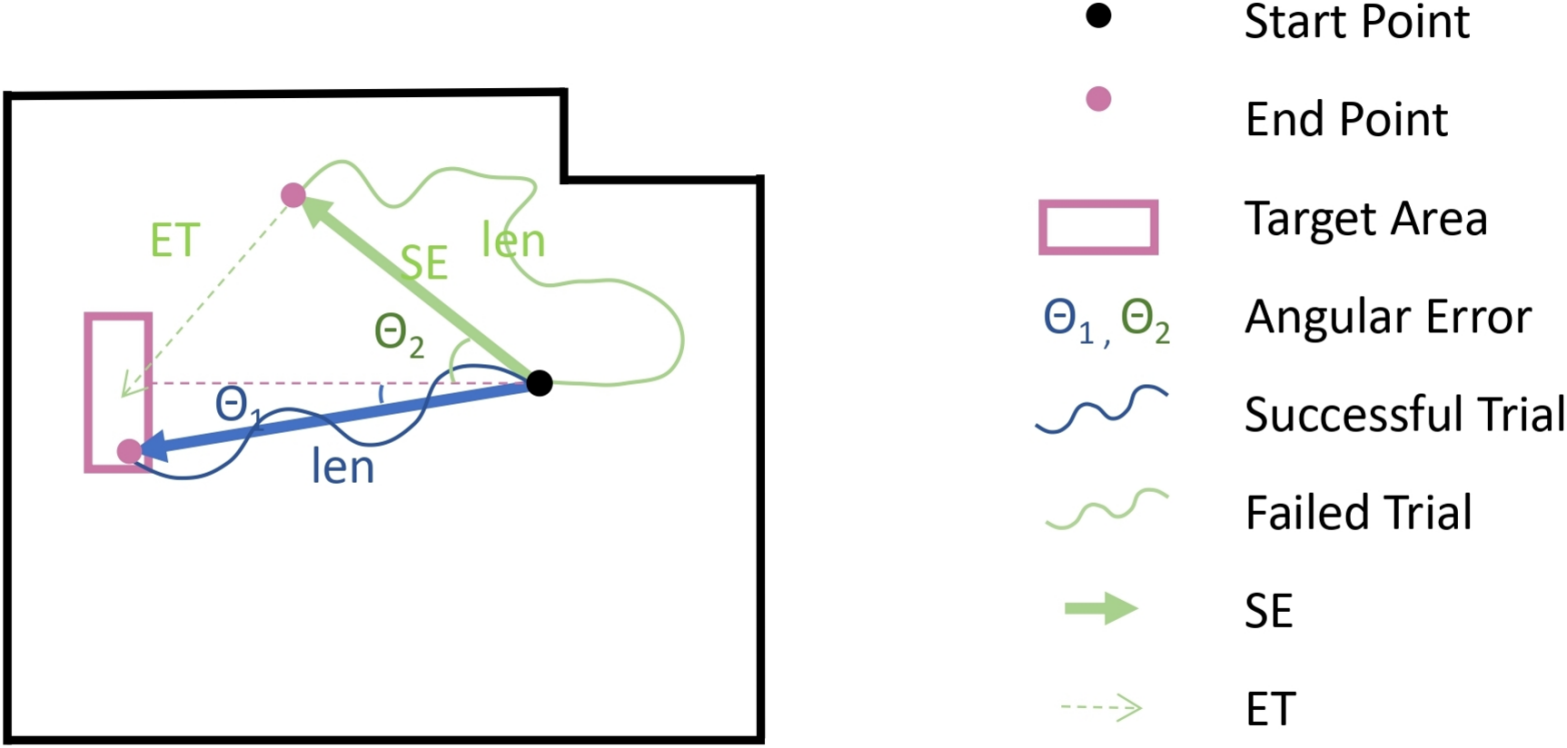
Measures of performance. For each trial, success is when the FOV arrives in the target area, fish gets food reward. Angular error is the angle between the direction form start point to end pint and the direction form start point to target point. Distance traveled is the total trajectory length of the successful trials. For failed trials, distance traveled is the total trajectory length plus the distance form end point to target point multiply efficiency (see Section 2) of the fish in the session. SE, the distance from start point to end point. ET, the distance from end point to target point.

The raw data consist of the position in the arena provided by the LIDAR and the position and posture of the fish in the tank provided by the onboard camera. We set Time 0 to be the moment at which the fish appeared in the frame after the buckets was removed. Frames where no fish were detected were removed from analysis and later smoothed over. Then, based on the position information of the FOV, we can calculate some metrics of the FOV trace of each trial. In this paper, we characterize the performance of each trial based on three measures (Figure 2):

1. Success/session: The number of food pellets the fish gets in a session. This was tested by testing whether $\left(x_{-1},y_{-1}\right)$ was in the target area (where $\left(x_{-1},y_{-1}\right)$ is the final data point collected for the trial). The maximum success/session is 6 because there are six trials in each session.
2. Angular error: the angle between the fish's actual trajectory and the ideal trajectory (measured at the end point). That is, using ($x_{0},y_{0}$) to indicate the first data point collected on each trial. The optimal angular error is 0°.
$$\text{angular error}=\arctan\left(\frac{y_{-1}-y_{0}}{x_{-1}-x_{0}}\right)$$
3. Distance traveled: The adjusted trajectory length calculation considers two possibilities. For successful trials, it is simply the actual trajectory length (solid blue line). For unsuccessful trials, the actual trajectory length is increased by an estimate of the additional trajectory that would be required to reach the target. This is done using $\left(x_{t},y_{t}\right)$ to indicate the current data point collected on the trial and $\left(x_{t-1},y_{t-1}\right)$ to indicate the previous data point collected on the trial. Optimal distance traveled is 2.5 m from origin to target.
$$\text{length}:\;\mathrm{len}=\sum_{t=1}^{-1}\sqrt{{\left(x_{t}-x_{t-1}\right)}^{2}+{\left(y_{t}-y_{t-1}\right)}^{2}}$$

$$d=\left\{\;\begin{array}{cc} \mathrm{len} & \text{if trial successful}\\ d+\mathrm{EAL} & \text{otherwise} \end{array}\right.$$

The adjusted length includes an estimated additional length (EAL) to compensate for the fact that unsuccessful trials can end far from the target. The EAL estimates the additional distance that would be traveled from the end of the trial to the target assuming the same efficiency as the fish has shown in other trials of that session. That is,

$$\text{distance from start to}\;\mathrm{end},\mathrm{SE}=\sqrt{{\left(x_{-1}-x_{0}\right)}^{2}+{\left(y_{-1}-y_{0}\right)}^{2}}$$

$$\text{distance from}\;\mathrm{end}\;\text{to target},\mathrm{ET}=\sqrt{{\left(x_{T}-x_{-1}\right)}^{2}+{\left(y_{T}-y_{-1}\right)}^{2}}$$

$$\text{efficiency of the session},\mathrm{eff}=\frac{\sum_{T=1}^{6}\mathrm{le}n_{T}}{\sum_{T=1}^{6}SE_{T}}$$

estimated additional length,EAL=eff×ET

For all measures, statistical analysis (described below) is performed at the level of individual trials. However, for presentation purposes, sessions are usually represented by the median across trials in that session.

Fish were moved from the baseline stage to the rotation stage when, on three consecutive days, the median performance of the 3 days included at least four successful trials, and the trials had median direction less than ± 15° and median length less than 4.5 m. Fish that did not reach this success criterion after 25 baseline sessions were removed from the experiment.

### 2.6 Statistical Analysis

The statistical results are based on a Bayesian regression. The model is described here in brief with detail provided in the Supporting Information and all code and even more information available in Zenodo hosted notebooks and git repository. We followed a modern Bayesian workflow (Gelman et al. 2020; Maresch et al. 2020; Martin et al. 2021), which includes five stages: model selection, prior selection, sampling (we skipped posterior predictive tests here), and interpretation of the posterior distribution. Further details of the model, including full parameter lists, choices of prior, prior predictive checks, sampling diagnostics, and posterior predictive checks, are all available in the Supporting Information. We explain the analysis by stepping through the different stages of the workflow and explain what was done in each one.

We report our results using the median and 94% HDI of the median. The HDI contains 94% of the distribution, in which every point has higher credibility than any points outside this range. Furthermore, for stage comparisons, we specified a region of practical equivalence (ROPE) of the groups. The ROPE for success is a difference of one success/session; the ROPE for the angular error is a difference of 5°; the ROPE for the distance is a difference of 2 m. These ROPEs are arbitrary, but we report the overlap of the posterior and the ROPE to give a general sense of the probability that differences are significantly different from 0. Unless otherwise indicated, brackets represent 94% HDI.

1 Model selection

We built our hierarchy models based on different measures of data. For each measure, the actual likelihood used was different—as appropriate for the different data types (Section S1). For the success/session (Figure S1, Section 2), we used a Poisson likelihood (so variance scaled with the median). For the angular error (Figure S2, Section 6), we used a Student's *t* likelihood. For the distance traveled, we used a Gamma likelihood (Figure S3, Section 7).

$$\text{Success}/{\text{Session}}_{i}∼\text{Pois}\left(\text{rate}=\hat{\mu }\right)$$

$${\text{Angular Error}}_{i}∼{\text{Student}}^{\prime }s\;t\left(\text{mean}=\hat{\mu },\mathrm{std}=\hat{\sigma },\mathrm{df}=\nu \right)$$

$${\text{Distance Traveled}}_{i}∼\text{Gamma}\left(\text{mode}=\hat{\mu },\mathrm{std}=\hat{\sigma }\right)$$

where ν is fixed across the data and has an exponential prior. The models assume that median performance on each session follows an exponential curve:

$$\hat{\mu }=A_{\mu }\exp\left(-\frac{\mathrm{ses}}{\tau_{\mu }}\right)+\mu_{\mu ,\infty }$$

The actual spread of the data around this exponential curve is itself also modeled as an exponential:

$$\hat{\sigma }=A_{\sigma }\exp\left(-\frac{\mathrm{ses}}{\tau_{\mu }}\right)+\sigma_{\mu ,\infty }$$

2 Prior selection

We chose prior distributions that were appropriate to the nature of the parameter. Positive parameters use a Gamma prior, continuous parameters use normal priors, and the degrees of freedom use an exponential prior (which is traditional). Priors were generally determined in the spirit of empirical Bayes. Where possible, the center of the prior was set to the most reasonable value that could be guessed from the data. Then, the width of the prior was chosen to be broad enough to allow for unbiased estimates. We tuned the priors by sampling prior predictive distributions and changing the parameters to get datasets that include all reasonable data and exclude pathological possibilities (Figure S4).

3 Sampling

We generated MCMC samples from the joint posterior distribution of the parameters using the PyMC Python package (5.12.0). Much of the initial analysis and presentation uses the Python package Arviz (0.18.0). We used 4 chains, 1000 tune, and 1000 draw (8000 total) samples. We used standard diagnostics to ensure that the chains converge to a unimodal distribution for all parameters and that the results are consistent across chains.

4 Interpretation of the posterior distribution

Once the model was fit to the data, we used the regression median as our basis for comparing performance across fish. In addition to presenting the learning curve for each measure, we also looked at three additional parameters to characterize fish learning:

$$\text{initial value}=A_{\mu }+\mu_{\mu ,\infty }$$

$$\;\text{asymptotic performance}=\mu_{\mu ,\infty }$$

$$\text{initial learning rate}=-A_{\mu }/\tau_{\mu }$$

For each of these parameters, we calculated the HDI from the posterior distributions for each fish in each stage. Considering our model assumptions, we compared the values across fish and across stages.

## 3 Results

The experiments aimed to reveal adaptation of swimming pose in goldfish. The FOV is a custom‐built apparatus designed to allow a fish to control its movement within a defined arena (Figure 1A,B). Fish received baseline training to control the FOV and drive it from a fixed origin to reach a pink target and receive a food reward (Figure 1C). Then, the fish were subjected to rotation of the control of the vehicle such that the fish command to the vehicle was rotated by 45° for 15 sessions (Figure 1D). Following the rotation sessions, the perturbation was removed for 10 sessions to test for washout. Finally, the fish were subjected to an additional savings stage (15 sessions) and a final washout (10 sessions). Using this experimental timeline, we can explore the characteristics of fish learning, aftereffects, and relearning (Figure 1E). Of the 12 fish that began the experiment, 8 completed the baseline (3 of these died during baseline, and 1 did not succeed in achieving the baseline performance criteria).

### 3.1 Goldfish Can Operate the FOV

**FIGURE 3:**
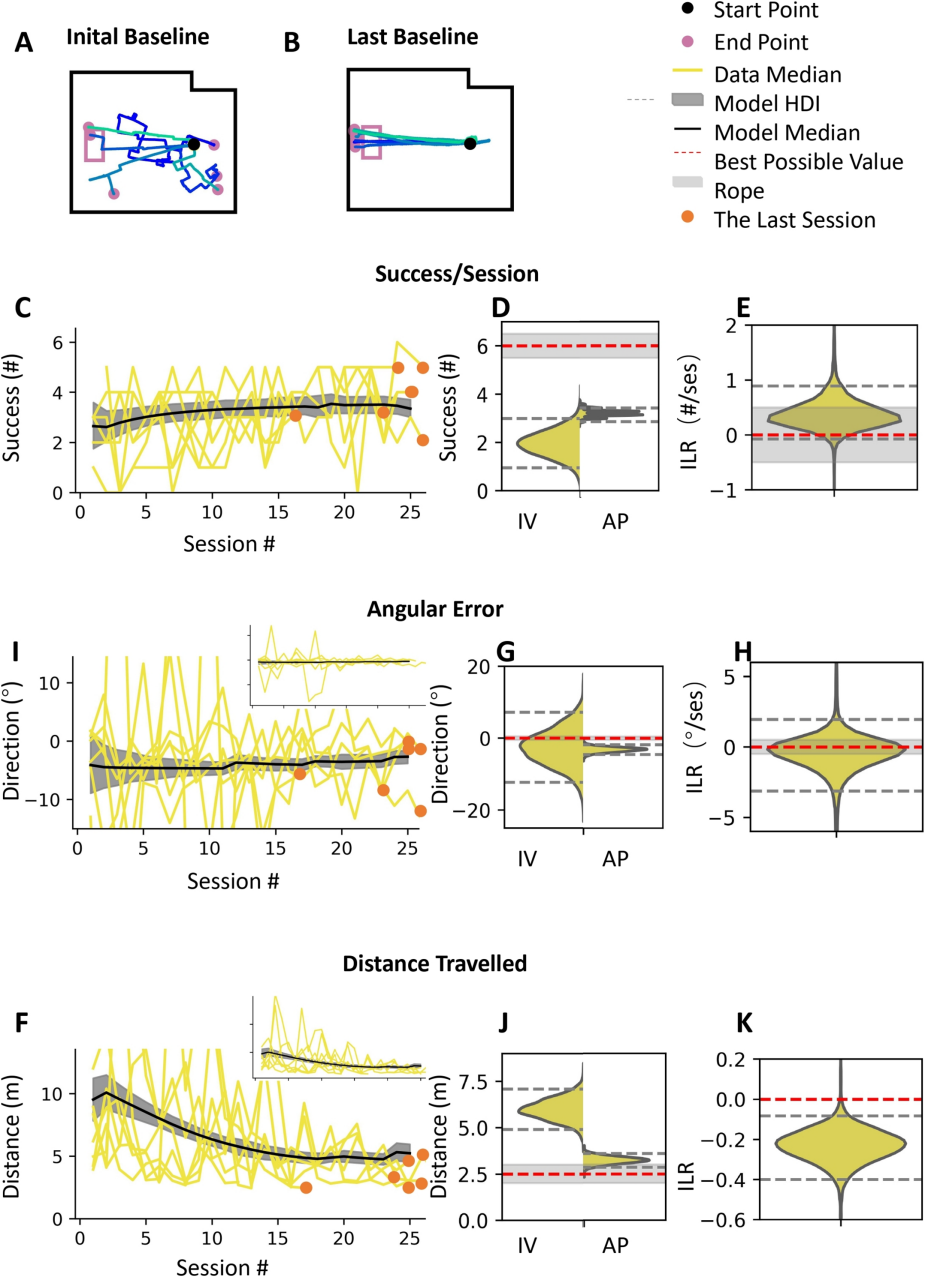
Goldfish can operate the vehicle. (A, B) The trace plots of the example fish. (C–E) Data and model parameters of success/session. (H, I) Data and model parameters of angular error. (F–K) Data and model parameters of the distance traveled. (C, I, F) Data of median value of each fish performance. The yellow lines are the medians of the measure of the fish in one session. The orange points are the last baseline sessions of the fish. The black line is the model estimation of the median performance across the fish. The gray patch is highest density interval (HDI) of the model estimation of the median performance. (D, G, J) Parameters we got from the model fit. IV, initial value of the performance. AP, asymptotic performance. We can compare the initial performance and asymptotic performance by these figures. (E, H, K) ILR. Initial learning rate, the learning rate of the first session. These plots reflect how fast is the motor adaptation of the FOV experiment.

Goldfish can drive the FOV, corroborating Givon et al. (2022). In Figure 3A, initial baseline movements of the example fish can be seen to be unsuccessful, where late baseline movements generally reach the target; the direction also improves, and although some early baseline movements are short, when the length is adjusted for the distance from the target, the fish has clearly improved by the late baseline (Figure 3B). Fish show improvement in all measures during baseline.

Figure 3C shows the success of the fish over all baseline trials. We calculated the high‐density‐intervals (HDI) form the posterior distributions for all stages. Across fish, the asymptotic performance improved from initial value 1.9 success/session (HDI 0.9–2.9) to 3.2 success/session (HDI 3.0–3.4, Figure 3D). The angular error became more consistent with an HDI that goes from −2.6° (HDI −12.5° to 6.9°) to −3.2° (HDI −4.5 to −1.9°) (Figure 3G), and distance traveled improved from 6.0 m (HDI 4.9–7.0) to 3.2 m (2.9–3.6) (Figure 3J). The baseline was completed in a median of 24 sessions (minimum: 12; maximum: 25). All the fish who completed the baseline went on to train in the rotation. Additional fish died along the way and the number of fish providing data for each stage was washout, 7 fish; saving, 7 fish; and final stage: 3 fish.

### 3.2 Goldfish Can Adapt to Rotation in Operating the FOV

In the rotation stage, we added a 45° perturbation between the mapping of the fish's orientation and the vehicle's direction of movement (as described in Section 2). The objective of this rotation was to test if the fish could learn from the errors caused by the changed mapping. If the fish continues swimming towards the target, this will cause a 45° error in direction. This may also increase the length of the movement as the fish struggles to reach the target and may reduce the number of successes if the fish does not ultimately reach it. This situation is expected in the early rotation stage.

We hypothesized that fish will eventually adjust their orientation to counteract the effects of the perturbed mapping, causing the vehicle to go straight to the target again. If, on the other hand, the fish cannot learn the new control condition, we expect success to stay low, the size of the directional error to stay large, and potentially also the length of the movement to stay long.

**FIGURE 4:**
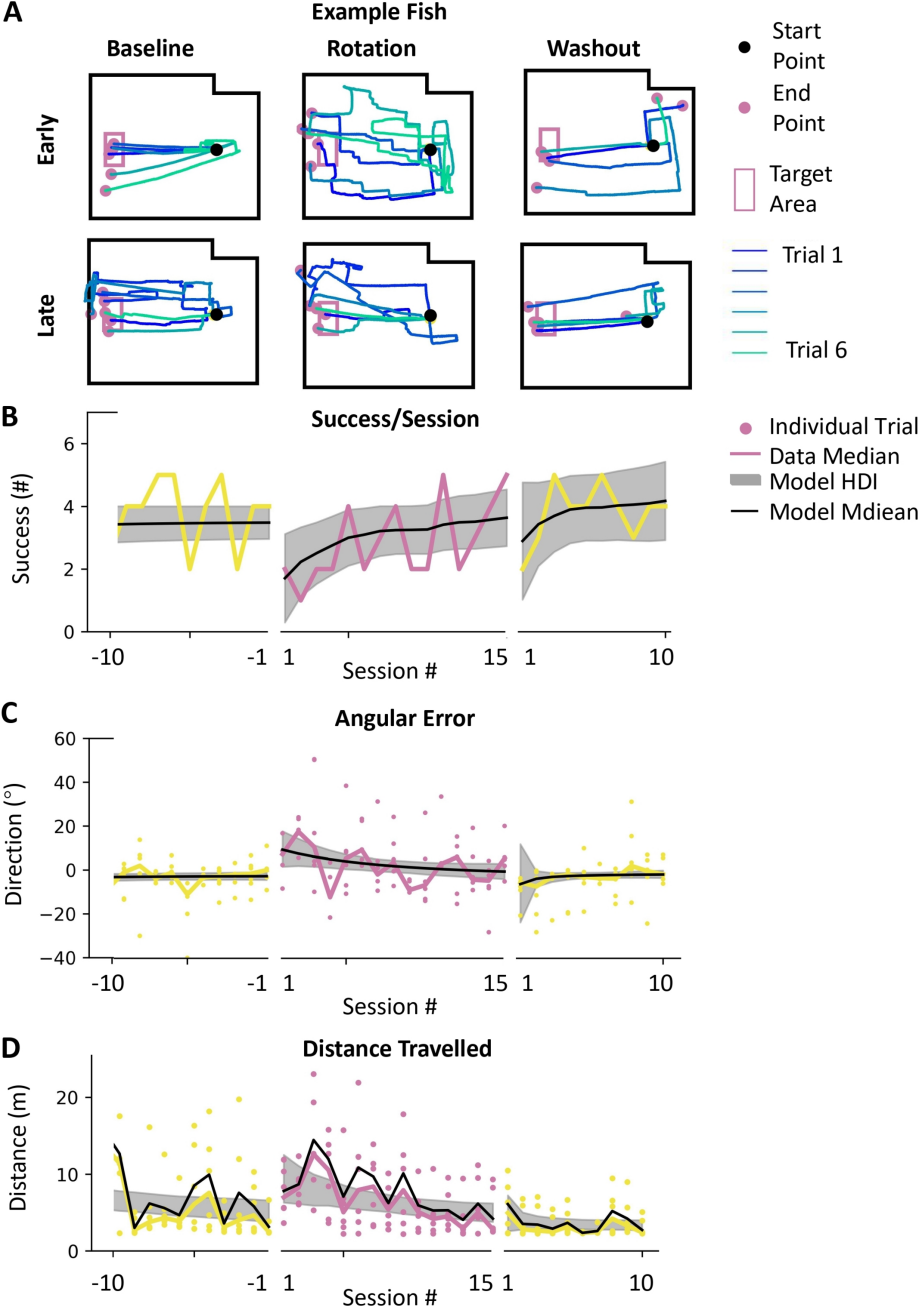
Goldfish can adapt to rotation. (A) The trace plots of the example fish. (B–D) The colorful lines are the raw median data in one session of the performance of the example fish. The colorful points are the data from individual trial. The black lines are the model estimations of the median performance of the example fish. The gray patches are HDI of the model estimation of the median performance.

The first two columns of Figure 4 allow comparison of baseline performance to performance in the rotation session. The data show that the example fish adapts to the rotation perturbation. The success improves from 1.4 to 3.7 success/session, angular error improves from 9.4° to −0.5°, and distance traveled improves from 6.4 to 3.7 m (Figure 4B–D). These numbers are the estimated median from the model fit to the fish data, with the actual data values shown in the figure.

We can characterize the fish's adaptation using the initial learning rate and asymptotic performance. In this fish, for instance, the initial learning rate was an increase of 0.5 successes per session, a 2.0° improvement in median angular error per session, and a 0.5‐m improvement in distance traveled. The asymptotic performance values were 3.6 successes/session, −2.7° angular error*,* and a distance traveled of 3.2 m (compared to an optimal length of 2.5 m from origin to target).

**FIGURE 5:**
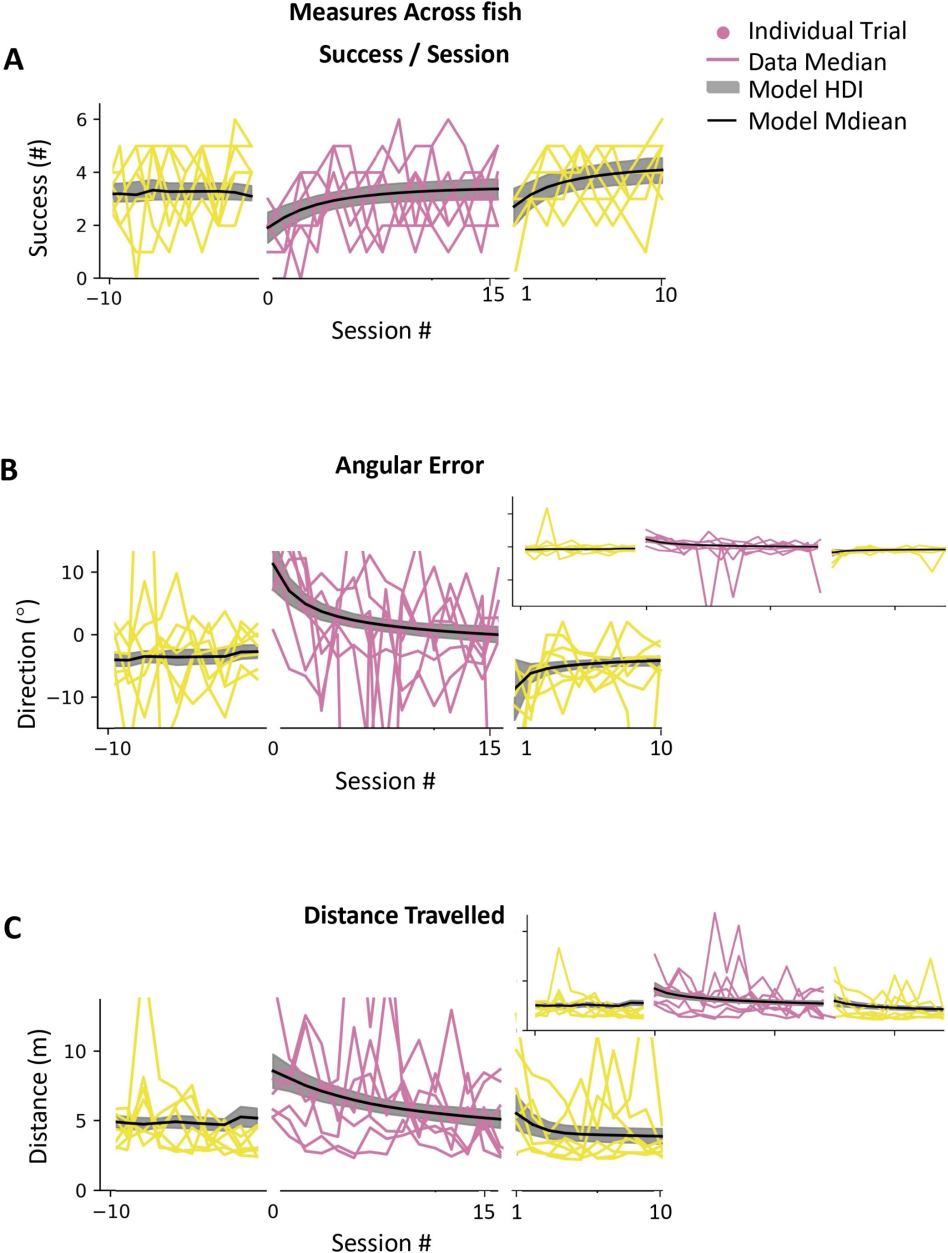
Goldfish can adapt to 45° rotation. (A–C) The colorful lines are the raw median data in one session of the performance of across fish. The black lines are the model estimations of the median performance across fish. The gray patches are HDI of the model estimation of the median performance across fish.

The performance across 45° fish is shown in Figure 5. The data show that across fish, we can see adaptation to the rotation perturbation. In this figure, each colored line is the median of the data for a fish, and the black line is the model estimate of the median across fish (the gray patch represents the HDI). The median success improves from 1.9 success/session (HDI 1.3–2.5) to 3.4 success/session (HDI 3.0–3.8), angular error improves from 11.4° (HDI 7.5°–15.8°) to 0.1° (HDI −1.3° to 1.3°), and distance numbers are the estimated median and the HDIs as taken from the model fit to the fish data, with the actual data values for individual fish shown with colored lines in the figure. Although the HDIs for all the measures overlap across fish, indicating some uncertainty, they have clearly moved in the expected direction, indicating fish have a general improvement in performance.

This improvement is also captured in the initial learning rate. Although the HDI of initial learning rate for each individual fish did overlap 0, the HDI for initial learning rate across fish for all measures did not. The initial learning rate was 0.58 success/session (HDI 0.1–1.1), −6.53° (HDI −13.1° to −1.8°) for median angular error per session, and −0.9 m (HDI −1.9 to −0.2 m) for distance traveled. Note that positive learning for success and negative learning for direction and length all indicate improved performance. Asymptotic values for all measures show a return to values near those at the end of baseline (success/session: rotation 3.5 success/session compared to baseline 3.2 success/session; angular error: −1.5° compared to −3.2°; distance traveled: 3.8 m compared to 3.2 m).

**FIGURE 6:**
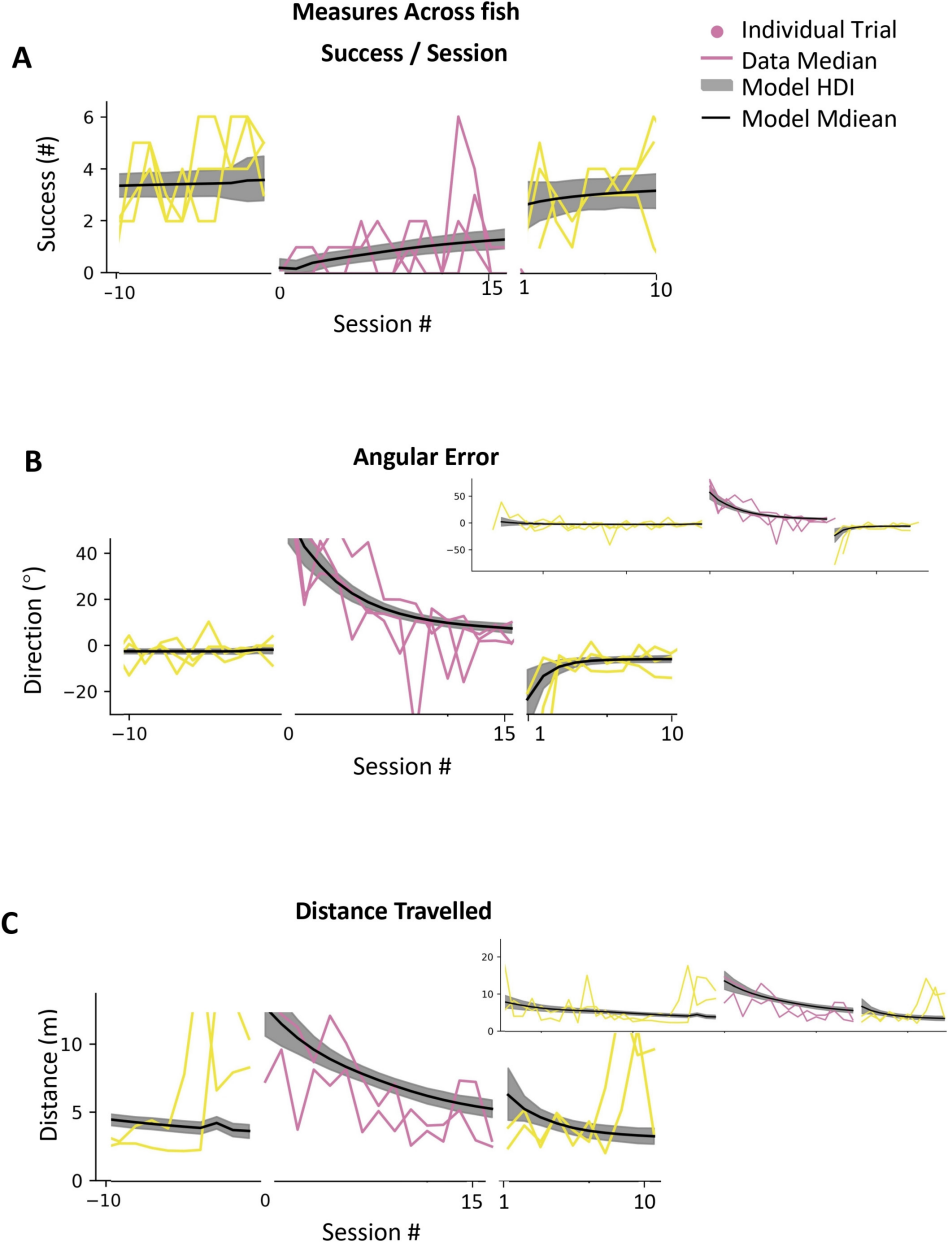
Goldfish can adapt to 90° rotation. (A–C) The colorful lines are the raw median data in one session of the performance of across fish. The black lines are the model estimations of the median performance across fish. The gray patches are HDI of the model estimation of the median performance across fish.

Figure 5 also shows after‐effects in all measures across all fish. Success/session initially dipped to 3.0 success/session (HDI 1.0–5.0), angular error showed a negative bias of −6° (HDI −17.2° to 3.3°), and distance traveled temporarily increased to 3.6 m (HDI 1.7–5.6) before returning to baseline levels (success/session: initial washout 3.5 compared to late baseline, 3.4; direction: −7.0° compared to −3.1°; length: 3.6 m compared to 3.4 m). The existence of aftereffects indicates the fish had formed an internal model of the rotation. Figure 6 shows the performance of 90° perturbation group. The median success during rotation sessions improves from 0 success/session (HDI 0.2–0.5) to 1.3 success/session (HDI 1.0–1.7), angular error improves from 57.0° (HDI 44.2°–70.2°) to 7.3° (HDI 5.3°–9.3°), and distance traveled improves from 11.6 m (HDI 10.0–11.6) to 4.6 m (HDI 4.1–5.0). The performance of −45°group is presented in Figure S8.

Thus, goldfish adapt to changes in their control environment and exhibit both motor adaptation and aftereffects.

### 3.3 In Savings Stage, Performance Improves but There Is No Classical Savings

In motor learning, “savings” refers to faster or improved relearning of a previously acquired motor skill. To investigate whether goldfish exhibit savings in whole‐body motor adaptation, we exposed them to the same perturbation a second time, comparing their performance in the initial rotation stage to a subsequent “savings” stage.

**FIGURE 7:**
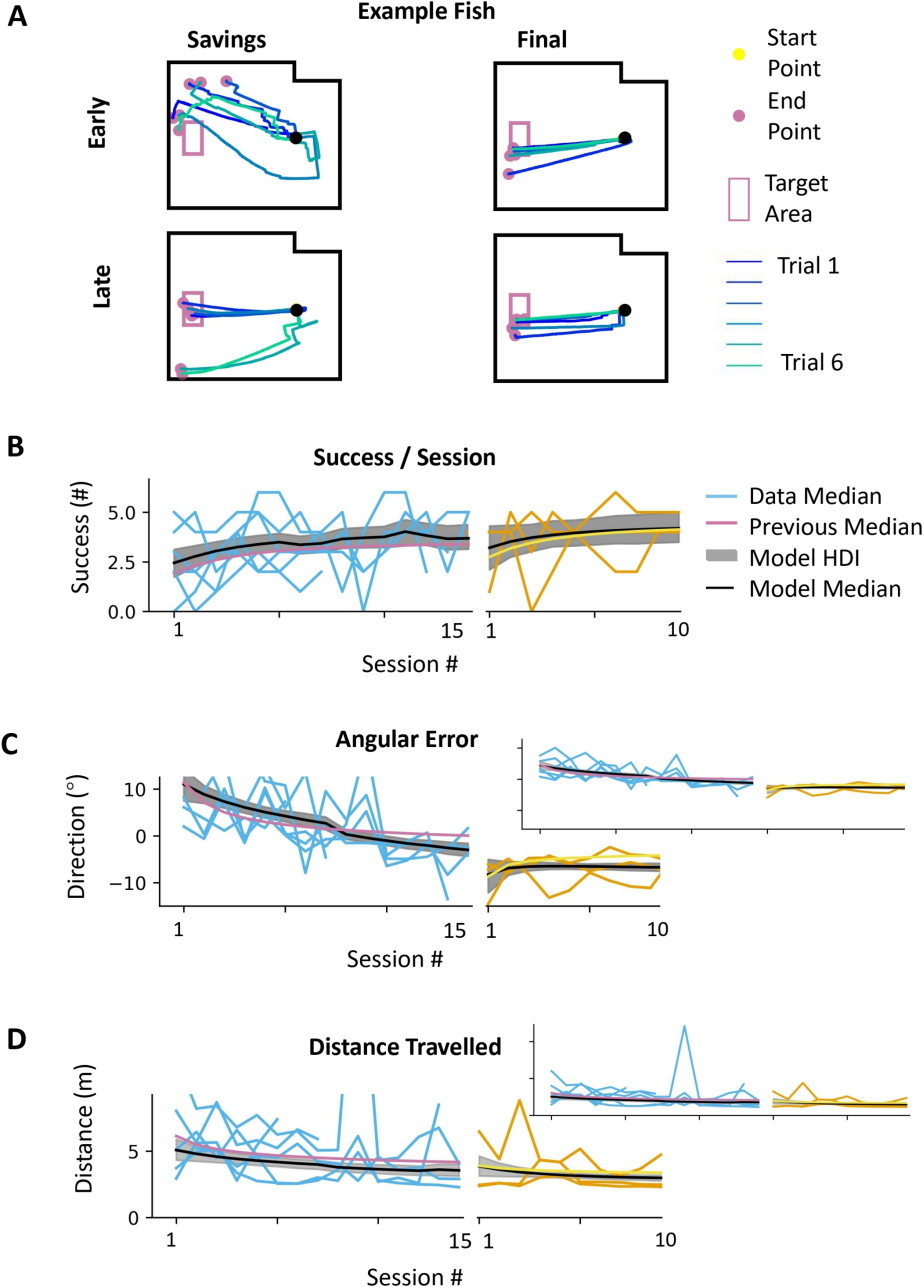
Slight improvement in performance in saving stage. (A) The trace plots of the example fish. (B–D) The sky blue lines are the median performance in each saving session. The orange lines are the median performance in each final session. The reddish‐purple lines are the model estimations of the median performance across in the rotation stage. The yellow lines are the model estimations of the median performance across in the washout stage. The black lines are the model estimations of the median performance across fish. The gray patches are HDI of the model estimation of the median performance across fish. The plots are the comparison of the performance measures between the rotation stage and saving stage.

**FIGURE 8:**
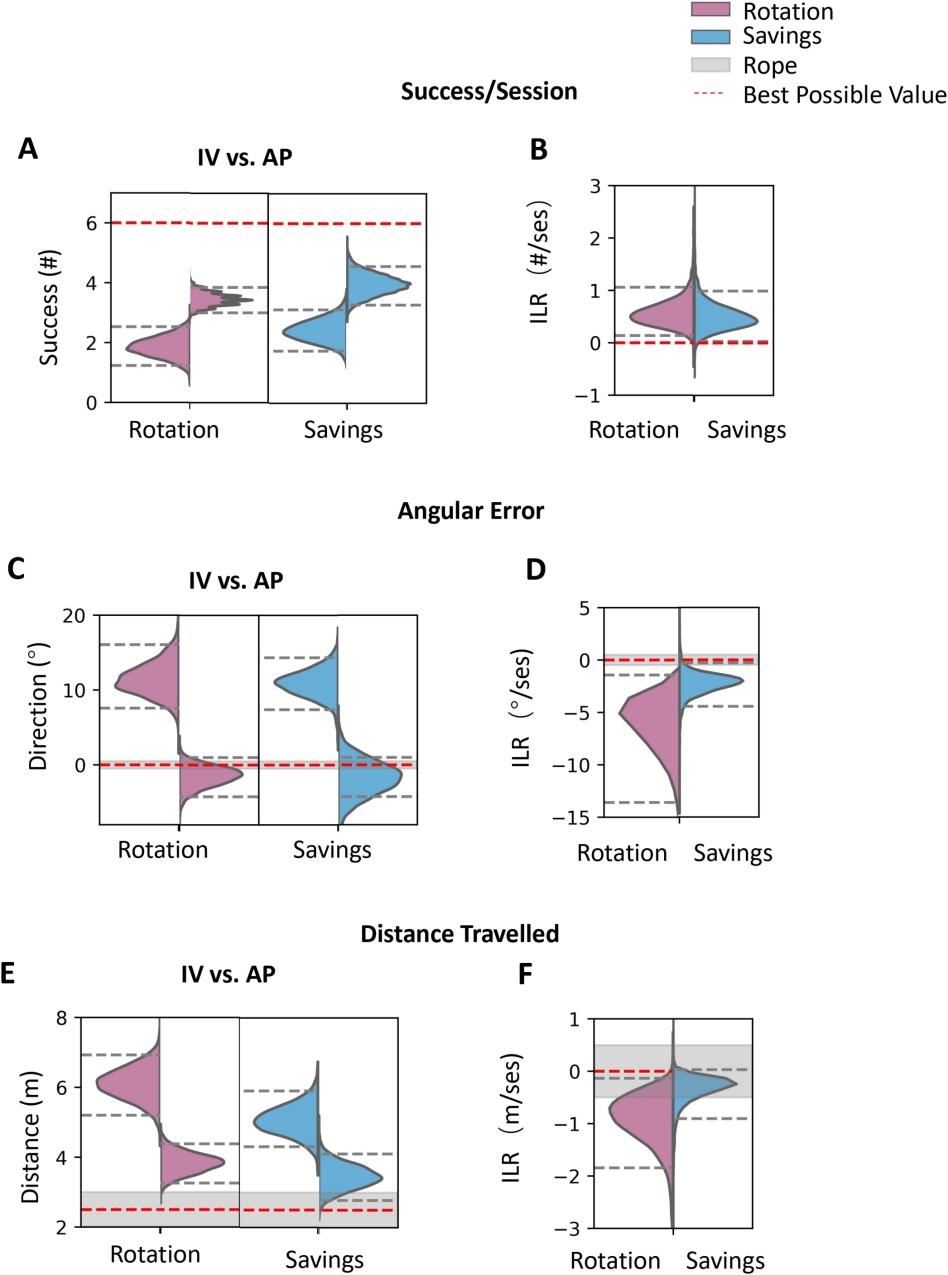
Slight improvement in performance in saving stage. (A,B) The parameters of success/session model. (C,D) The parameters of angular error model. (E,F) The parameters of distance traveled model. (A,C,E) IV (initial value) versus AP (asymptotic performance) in rotation and savings stage. (B,D,F) Comparisons of ILR (initial learning rate) in rotation and savings stage.

The performance across fish is shown in Figure 7B–D. We see that fish adapt to the second rotational perturbation. Success improves from 2.4 success/session (HDI 1.8–3.1) to 3.7 success/session (HDI 3.2–4.3), angular error improves from 11.0° (HDI 7.7–14.7) to −3.0° (HDI −4.3 to −1.6), and distance traveled improves from 5.1 m (HDI 4.2–5.9) to 3.6 m (HDI 3.1–4.0). Across fish, the learning rate in savings was 0.5 success/session (HDI 0.0–1.0), −2.4° (HDI −4.7° to −1.8°) for median angular error per session, and −0.4 m (HDI −0.9 to 0.0 m) for distance traveled. Similarly, asymptotic performance values were near those in baseline. The comparison of rotation and savings is shown in Figure 8 (success/session: initial learning rate of savings 0.5 compared to learning rate in rotation 0.6; angular error: −2.4 compared to −6.5; distance traveled: −0.4 compared to −0.9).

Thus, a split picture emerges. On the one hand, initial values in the savings session are better on all measures. This difference is small, well within the HDI, but it is consistent across measures. This reflects some level of savings. On the other hand, the learning rate in the savings session is actually smaller than the learning rate in the rotation session. This is not what we expected. However, the lower learning rate in the savings may, in fact, be related to the improved initial performance. Because initial performance in savings is better, there is simply less to learn.

In conclusion, despite showing better relearning performance in some measures during the savings stage, goldfish did not demonstrate significantly faster initial learning rates compared to the initial rotation stage. Therefore, we conclude that goldfish showed no clear evidence of savings in this whole‐body motor adaptation task.

### 3.4 Negative Correlation Between the Learning Rates in Perturbation Stages

Examination of the data underlying Figure 8 reveals a weak correlation between learning rates in the initial rotation and subsequent rerotation (savings) that is consistent across measures. The slope correlation between these two measures across fish is −0.327 (HDI −1.7, 0.07) for success/session,−0.193 (HDI −0.7 to 0.1) for angular error, and −0.172 (HDI −0.7 to 0.2) for distance traveled.

In conclusion, these findings highlight the complexity of motor learning in goldfish and emphasize the importance of considering individual variability in such studies. The observed patterns suggest that the fish's prior learning experience influences their subsequent learning potential, although not necessarily in the form of traditional savings.

## 4 Discussion

We tested whole‐body motor adaptation in goldfish while they operated the FOV. After training the fish to drive to a target area (Figure 3), we introduced a perturbing mapping between their orientation and the FOV movement. Initially, the fish failed to perform the task, and the trajectory error was large. Then, over the course of several sessions, all fish that completed baseline training successfully adapted to operate the FOV to the target, demonstrating their ability to adapt to the rotated mapping (Figure 5). Following the removal of the perturbation, we observed an aftereffect in which the fish made errors in the opposite direction (Figure 5). This result suggests the formation of an internal model in the fish's brain that anticipates perturbation.

We also investigated whether goldfish exhibit savings in adaptation when facing the same perturbation a second time. Following the washout session, we reintroduced the same perturbation to the mapping (Figure 7). Although the fish showed better overall performance relative to the first rotation stage, we did not observe a significant difference in learning rate (Figure 8). This indicates that the fish did not show a faster adaptation. However, we do see better performance in initial value and asymptotic performance across all the measures.

There was a negative correlation in all three measures between learning rate in the initial rotation and in the savings. This suggests that fish that learn efficiently in the initial stage may have less room for improvement in subsequent exposures, whereas those that struggle initially have more potential for improvement in later learning phases.

In previous studies, fish have been shown to exhibit some basic properties of motor adaptation. For instance, Yang et al. (2024) discovered that electric fish learn to retune their controllers to compensate for destabilizing dynamics and retain their learned controllers, showing a clear aftereffect. Similarly, Volotsky et al. (2024) demonstrated the archerfish's ability to adapt its shooting to environmental changes, also observing an aftereffect after perturbation removal. However, these studies did not include a relearning stage after the washout stage, which is common in human motor adaptation studies. We did not see a clear savings effect, and this may suggest differences in motor adaptation mechanisms between fish and mammals (D'Elia and Dasen 2018; Lucon‐Xiccato et al. 2017; Spampinato and Celnik 2021).

There are several motor control studies that, either directly or indirectly, examined visual motor and subsequent postural compensations of zebrafish (Ahrens et al. 2012; Christie and Severi 2021; Xu et al. 2021). And in human studies (Ahmed and Wolpert 2009; Babič et al. 2016; Bakkum et al. 2020), there is support for the existence of separate mappings for posture and movement, which adapt independently but encode similar dynamics. In spite of the use of the optomotor response to study the motor adaptation of fish eye movements (Aksay et al. 2007; Beck et al. 2004; Major et al. 2004), it is time to test these computational ideas with the swimming posture of fish. With this motivation, we designed an experimental paradigm that exposes sensorimotor control mechanisms and the adaptation to the continuous water disturbance. We studied an unconstrained whole‐body motion where the fish subjects performed a specific posture swimming behavior that was methodologically subjected to nontrivial perturbations.

The use of the FOV represents a novel approach to studying fish locomotion, allowing for the creation of virtual flows through manipulation of its control system. This method avoids the need for man‐made water flows to disturb the fish (Jindrich and Full 2002; Liao 2007; Webb 2004), providing a new tool for investigating motor adaptation in aquatic organisms. However, it also raises important questions about the ecological validity of the task. The fact that some fish did not learn to operate the FOV in the baseline session suggests that the task may not be equally suitable for all individuals, pointing to the need for further research on individual differences in learning ability and their potential impact on adaptation.

In conclusion, our study employed a novel methodology and a motor adaptation framework to investigate the self‐correction process in goldfish. This approach sheds light on motor adaptation in vertebrates' free‐swimming behavior in altered flows, potentially opening new avenues for comparative studies of adaptation across species and taxa. Future research could focus on exploring the neural mechanisms underlying these differences in motor adaptation between fish and mammals, as well as investigating whether other fish species show similar patterns of adaptation and lack of savings.

## Author Contributions

**Zhuoxin Liu:** conceptualization, data curation, formal analysis, investigation, methodology, visualization, writing – original draft. **Shachar Givon:** conceptualization, data curation, formal analysis, investigation, methodology, supervision, visualization, writing – review and editing. **Ronen Segev:** conceptualization, formal analysis, funding acquisition, investigation, methodology, project administration, resources, supervision, validation, visualization, writing – review and editing. **Opher Donchin:** conceptualization, formal analysis, funding acquisition, investigation, methodology, project administration, software, supervision, validation, visualization, writing – review and editing.

## Conflicts of Interest

The authors declare no conflicts of interest.

## Peer Review

The peer review history for this article is available at https://www.webofscience.com/api/gateway/wos/peer‐review/10.1111/ejn.70241.

## Supporting information


**Data S1:** Supporting Information.

## Data Availability

The csv data generated during and/or analyzed during the current study are available in the data of the Bayesian model figshare repository with the identifiers (https://doi.org/10.6084/m9.figshare.29626826). The code is archived in Zenodo (https://doi.org/10.5281/zenodo.16367403).
