# Supplementary material for: Whole Body Motor Adaptation in Goldfish Using Fish Operated Vehicle
Source: Eur J Neurosci. 2025 Sep 2;62(5):e70241. doi: 10.1111/ejn.70241 (PMC12404787; doi:10.1111/ejn.70241)
Supplement: Supplementary file 1 — Data S1: Supporting Information. [file EJN-62-0-s001.pdf]

Description of the statistical model

We fit three different Bayesian regression models for the three measures used to evaluate the performance of the fish. These are all hierarchical models of similar form, with differences due to the distinct data types of each measure. Success per session is discrete or count data, angle is a continuous variable, and distance is positive. We followed a modern Bayesian workflow (Gelman et al. 2020; Maresch et al. 2021), which includes five stages: model selection, prior selection, sampling, posterior predictive tests, and interpretation of the posterior distribution. While this description of the model and analyses is thorough, the full details— including all prior choices, prior predictive checks, full sampling diagnostics, and posterior predictive checks— are in our Jupyter notebook. Our goal here is to explain the analysis by stepping through the stages of the workflow, outlining what was done in each one, and providing representative figures to demonstrate the process.

Motor Learning Laboratory, Department of Biomedical Engineering, Ben-Gurion Uvniersity, Be'er Sheva, Israel. (2024, September 2). Motor-Learning-Lab/FOVsucmodel: submit
(Version 1.0.0) [Computer software]. Zenodo. [https://doi.org/10.5281/zenodo.13824664].

1. Model selection

The models described are the result of a process of model selection. A number of factors influenced our choices. First, we prioritized interpretability. That is, early on we made the choice to use an exponential fit so parameters of the exponential fit would have meaningful interpretations. This also drove the choice to parameterize all Gamma distributions with the model and standard deviation instead of rate and shape. Second, we wanted the models for the different measures to be as similar as possible. This was a challenge because the data type for each measure is different. It was key in the later decision to characterize all measures using the median rather than the natural parameters that control the likelihood. Third, we opted for simplicity where possible. This led us to models that were non-hierarchical and with relatively few parameters. There are some modeling choices that were the result of quality of fit. The choice to use a Poisson distribution, with only one parameter, rather than some two parameter variant was a result of reasonable fit with the Poisson. In the other direction, we used a t distribution for the angle rather than a normal distribution because we observed that the outliers made the fit very noisy with the normal distribution.

1.1 Success/session

The data consists of the number of successes in each session. Since the data is count-based and non-negative, it fits a **discrete positive distribution**. Given this, we decided to use a **Poisson likelihood**, which is commonly used for modeling count data. The Poisson distribution assumes that the variance increases with the median, which reflects the real-world behavior in this

measure.

The underlying assumption for the model is that the **median performance over sessions** follows an **exponential curve**. This means we expect performance to change in a non-linear fashion as the sessions progress, following either exponential growth or decay.

To estimate parameters of this exponential model, we used a **Bayesian statistical** approach. This combines prior knowledge (or assumptions) about the population (encoded in the prior distributions) with the observed data (the evidence) to generate a **posterior distribution** of the parameters. This approach allows for a more flexible estimation process compared to traditional frequentist methods.

The structure of the model is shown in Supplementary Figure 1. These models are most easily read from the bottom to the top: the data, y, is generated by a Poission distribution whose rate parameter, $\hat{\mu}$, is calculated using an exponential equation with three parameters: A_u is the gain constant, τ_u is the time constant, and $\mu_{u,\infty}$ is the asymptotic value at time infinity. Each of these has a prior as shown in the figure. The mathematical notation that is sometimes used to describe models also appears in the figure. Note that all Gamma functions in the model are parameterized with mode and standard deviation for interpretability. Since pymc parameterizes Gamma distribution using shape and rate (pymc.Gamma — PyMC dev documentation), we converted our chosen mode and standard deviation into shape and rate using standard formulas for this reparatemerization (Gamma function's mean and standard deviation through shape and rate - Mathematics Stack Exchange)

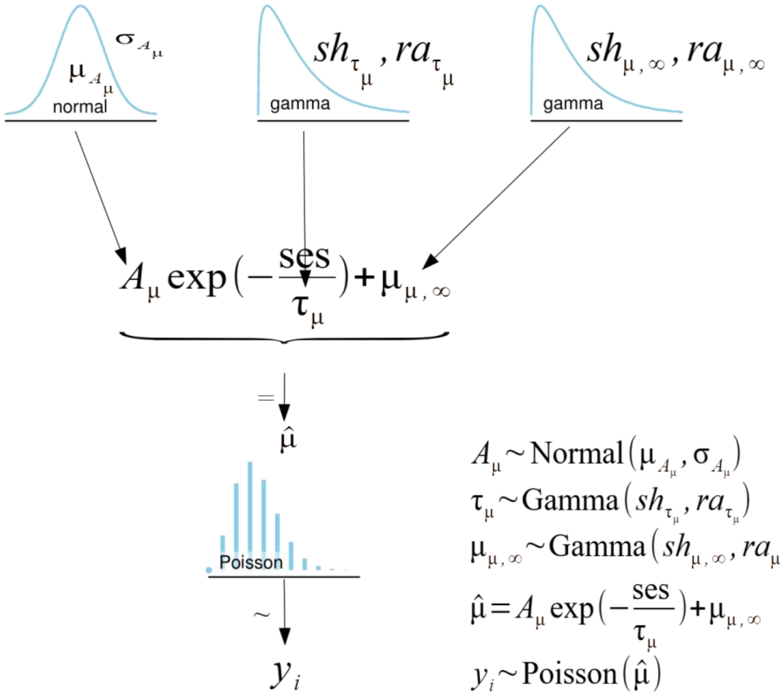

$$A_\mu \sim \text{Normal}(\mu_{A_\mu}, \sigma_{A_\mu})$$
$$\tau_\mu \sim \text{Gamma}(sh_{\tau_\mu}, ra_{\tau_\mu})$$
$$\mu_{\mu,\infty} \sim \text{Gamma}(sh_{\mu,\infty}, ra_{\mu,\infty})$$
$$\hat{\mu} = A_\mu \exp\left(-\frac{\text{ses}}{\tau_\mu}\right) + \mu_{\mu,\infty}$$
$$y_i \sim \text{Poisson}(\hat{\mu})$$

Supplementary Figure 1 The Bayesian model structure of success/session.

1.2 Angular error

The data consists of the angular error in each trial. Since the data ranges from 0 to 360°, it fits a **continuous distribution**. There were a few extreme values relative to the optimal value in the initial stages. Given this, we decided to use a **Student's t likelihood**, which forms a bell-shaped curve similar to the normal distribution but with heavier tails, allowing the t-distribution to better handle uncertainty.

Like with success / session, our model assumes that the **median performance over sessions** follows an **exponential curve**. We further assumed that the variance of the data around this median also follows an exponential curve. The structure of the model is shown in Supplementary Figure 2. The model is very similar to the model for success / session with two additions. First, using a t distributed likelihood for the data adds two additional parameters: the standard deviation, $\hat{\sigma}$, and the degrees of freedom, ν . As can be seen in the figure, $\hat{\sigma}$ is fit with an exponential decaying function like $\hat{\mu}$. ν has an exponential prior, which is the traditional choice.

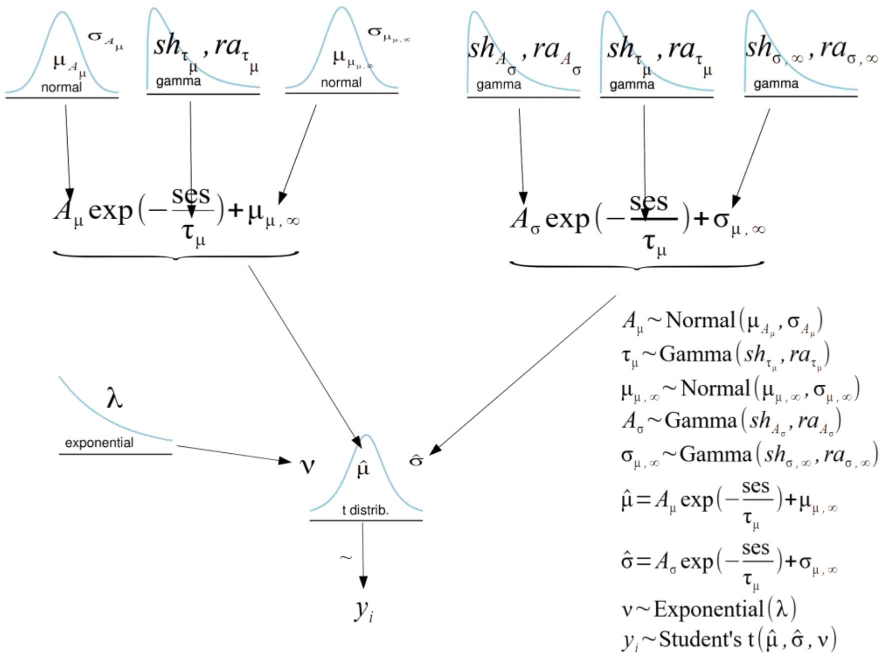

Supplementary Figure 2 The Bayesian model structure of angular error.

1.3 Distance travelled

The data consists of the distance traveled in each trial. Since the data ranges from 2.5 m to about 10 m, it fits a **continuous positive distribution**. Given this, we decided to use a **gamma likelihood**, which is well-suited for modeling positive values. The gamma distribution describes many real-life events, including predicted rainfall, the reliability of mechanical tools, or any application that only has positive outcomes.

As seen in Supplementary Figure 3, the modeling choices for this model mirror the ones made for the two previous models. One important modeling choice is that, as with all Gamma functions in the model, the likelihood is parameterized by the mode, $\hat{\mu}$, and the standard deviation, $\hat{\sigma}$. We use standard functions to reparameterize to the standard rate and shape parameters, but the exponential decay and hyperpriors are on the mode and standard deviation and not on the rate and shape.

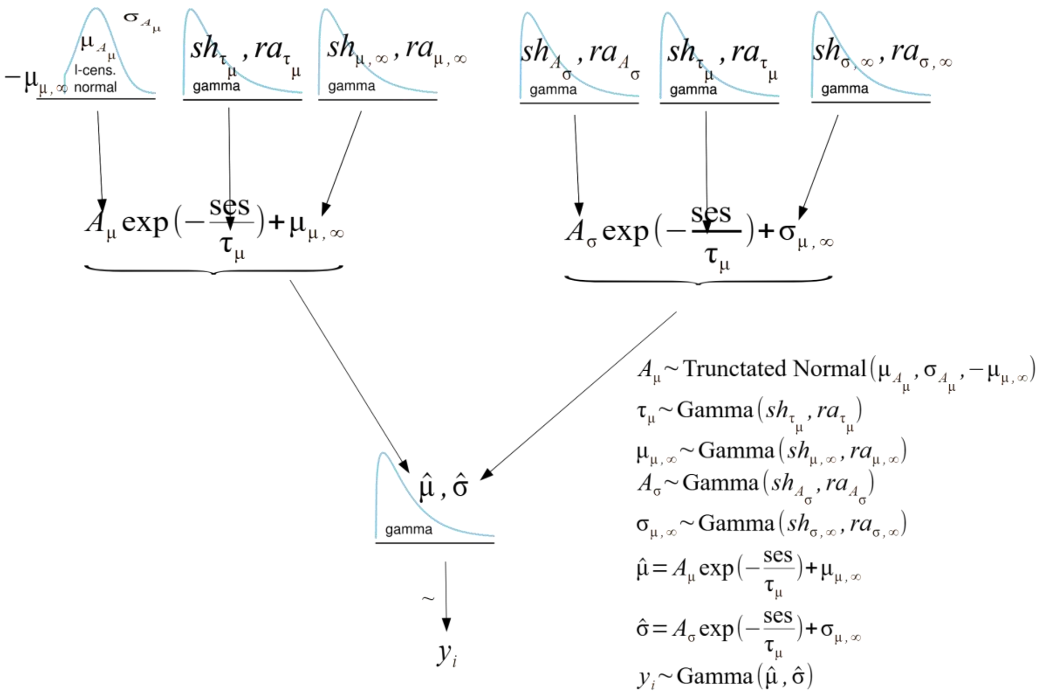

Supplementary Figure 3 The Bayesian model structure of distance travelled.

2. Priors

From this point, we focus on the model for success / session. The logic here will apply to the other models, and where there are differences, we point them out. Our general approach is sometimes called “empirical Bayes”, although we feel that this is a misnomer. That is, we used the data itself to set initial guesses about reasonable priors. We followed this up with prior predictive checks that see that the priors we chose led to broad but non-pathological data and iterated until reasonable priors were achieved.

2.1 Rationale for prior selection

The rate parameter ($\hat{\mu}$) of the Poisson distribution is central to the model, as it governs the mean and variance of the Poisson process. $\hat{\mu}$ is defined deterministically by the exponential model fit and thus we did not set priors directly on $\hat{\mu}$ but rather we set hyperpriors on the parameters that govern $\hat{\mu}$. The exponential fit has three hyperparameters: the amplitude (A_μ, or the amount of change between the first day and the learning asymptote), the learning asymptote, μ_∞, and the time constant, τ_μ.

It is worth noting that within our model, we applied consistent priors across all exponential curves for each fish, regardless of the learning stage. For each parameter, we compared the empirical values derived from the data to the final prior values used in the model, as detailed below.

Amplitude (A_μ):

We chose a Normal distribution for the prior of the amplitude (A_μ). This is a traditional choice for a continuous variable with no strict bound that can take positive or negative values. The amplitude reflects the difference between performance at the first and last sessions of each stage for each fish. We calculated the mean and standard deviation from the real data: we used the mean of the difference in performance across fish and stages in the last session and the first session as the mean of the normal distributions of each fish in each stage. Similarly, the standard deviation of this difference across fish and stages was used as an initial prior for the standard deviation. When we sampled the prior predictive using this prior, we found that the prior distributions μ_μ of some fish were negative. We adjusted the parameters to achieve positive prior predictive distributions. This led us to use 0.3*mean of the performance across fish on the last session across stages minus the first session (-0.61) as the mean and 2.08 (standard deviation of the performance across fish and stages of the difference between the last session and the first session) as the standard deviation as the prior of the normal distributions of each fish in each stage.

Asymptote ($\mu_{\mu,\infty}$):

The constant term $\mu_{\mu,\infty}$ represents the asymptotic value that performance reaches after many sessions. Since this value must be positive, we opted for a Gamma distribution. The prior for $\mu_{\mu,\infty}$ is informed by the performance at the last session of each stage for each fish. We used the mean of the performance across fish and stage on the last session as the mode of this gamma distribution. We used the standard deviation across fish and stage on the last session as the standard deviation. We then fine-tuned our priors based on sampling diagnostics, aiming to minimize divergences and improve model convergence. In the current version we used 3.63 (mean of the performance in the last session across fish and across stage as the mode of the gamma distribution of each fish in each stage - 0.6) as mode and 1.36 (standard deviation of the performance across fish on the last session across stage) as the standard deviation as the prior of the Gamma distribution.

Time Constant (τ_μ):

The time constant τ_μ determines the rate at which performance changes over time (i.e., how quickly the asymptote is approached). Like $\mu_{\mu,\infty}$, τ_μ must be positive, so we again used a Gamma distribution. The prior for τ_μ is based on the number of sessions that each fish experiences in a given stage. We calculated the mean and standard deviation using the number obtained from the last session minus the first session across fish across stage. In the current version we used 2.96 as mode and 2.24 as the standard deviation as the prior of the gamma distribution.

3. Prior predictive check

Prior predictive checks are a step in model building that precedes fitting the model to the actual data. A prior predictive check generates data from the prior distributions. The logic is that priors on the parameters are often difficult to interpret and that priors on different parameters can interact in unexpected ways. It is usually easier to judge whether priors are overly permissive or restrictive by determining what they imply about the data. A prior is overly permissive if it permits data which is obviously pathological. A prior can be overly restrictive in two ways: it may make the existing data highly unlikely or it may rule out all data except the existing data. In either situation, the prior will have an undue influence on the inference. By simulating data using these priors without incorporating the actual data, we can verify that they produce realistic values for parameters and outcomes, and inspect whether the model generates plausible results that resemble the real-world phenomenon we're modeling. This approach helps ensure that the chosen priors do not dominate the inference process and allows us to confirm that the results of our data analysis are dominated by the data.

When performing a prior predictive check, we're looking to confirm that the model can reasonably produce:

- Values of success/session that fall within a plausible range .
- Patterns in the simulated data that broadly match the expectations (i.e., how many successes/session of each fish would be expected purely from the priors)for how the system behaves.

In this case, the trial number for each session is 6, so the reasonable range of simulated data should be wider than 0 to 6 (Supplementary Figure 4).

We simulated the number of successes per session for each fish based solely on the priors. These simulated counts were then compared to the actual data to check for major discrepancies.

We displayed the distribution of simulated counts from the prior. These plots help determine whether the chosen priors are reasonable and whether they produce data that aligns with reality. This step ensures that the model will provide meaningful inferences once the actual data is incorporated.

4. Sampling

We generated MCMC samples from the joint posterior distribution of the parameters using the PyMC Python package (5.12.0). Much of the initial analysis and presentation uses the Python package Arviz (0.18.0). We used 4 chains, 1000 tune and 1000 draw (8000 total) samples. We used the standard diagnostics to ensure that the chains converge to a unimodal distribution for all parameters and that the results were consistent across chains .

For example, Supplementary Table 1 reports MCSE, ESS, Rhat value and Supplementary Figure 5 shows the trace plots and rank plots of A_{μ}, $\mu_{\mu,\infty}$, τ_{μ} of fish 9 in the rotation stage. Suppelmentary Figure 6 shows the partial autocorrelation plots. Interpretation of these plots is laid out in various introductory texts on Bayesian Analysis, such as pp. 40 - 51 of Bayesian Modeling and Computation in Python(Martin et al. 2021). For our purposes it is sufficient to say that they demonstrate that the sampling provides a reasonable characterization of the joint posterior distribution.

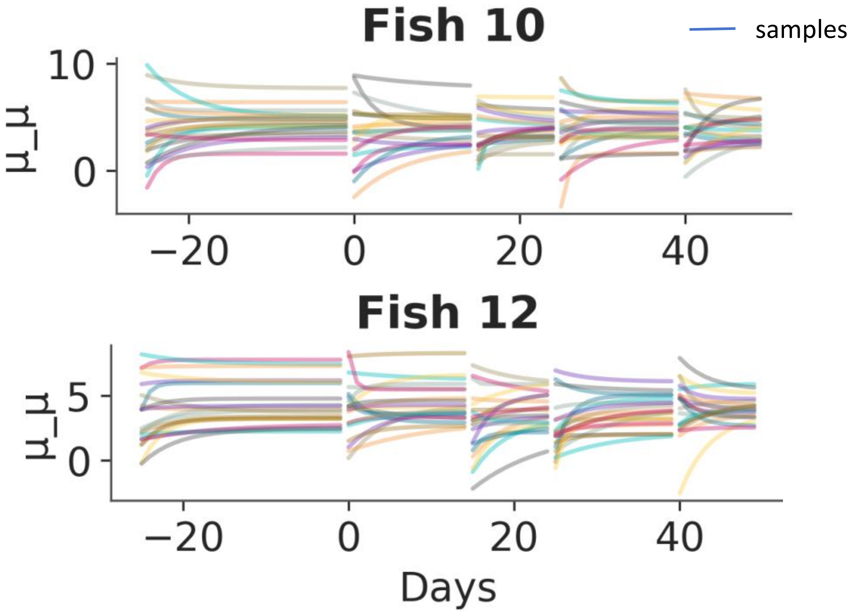

Supplementary Figure 4: Prior Predictive Checks of Success/Session Model for Two Fish. This figure displays prior predictive plots for two randomly selected fish (Fish 10 and Fish 12). Each plot shows 20 different random samples of simulated counts over time, with each color representing a distinct sample. The x-axis represents sessions (from -25 to 50), while the y-axis (μ_μ) represents the model's predicted success/session. In this case, the trial number of each session is 6. So the reasonable value of the simulated data should be about wider than 0~6.

	mean	sd	hdi_3%	hdi_97%	mcse_mean	mcse_sd	ess_bulk	ess_tail	r_hat
A_μμ	-1.102	0.992	-2.931	0.817	0.016	0.012	3736.0	3219.0	1.0
μ_μ_inf	2.973	0.547	2.018	3.993	0.008	0.006	4571.0	3008.0	1.0
τ_μ	5.363	3.016	0.675	10.725	0.043	0.034	4632.0	2593.0	1.0

Supplementary Table 1 : Legend: Rows: 'A_μμ': Amplitude parameter. 'μ_μ_inf': Asymptote parameter. 'τ_μ': Time constant parameter. Columns: 'mean': Posterior mean. 'sd': Posterior standard deviation. 'hdi_3%': 3% highest density interval. 'hdi_97%': 97% highest density interval. 'mcse_mean': Monte Carlo standard error of the mean. ' mcse_sd': Monte Carlo standard error of the standard deviation. 'ess_bulk': Effective sample size (bulk). 'ess_tail': Effective sample size (tail). r_hat: R-hat convergence statistic.

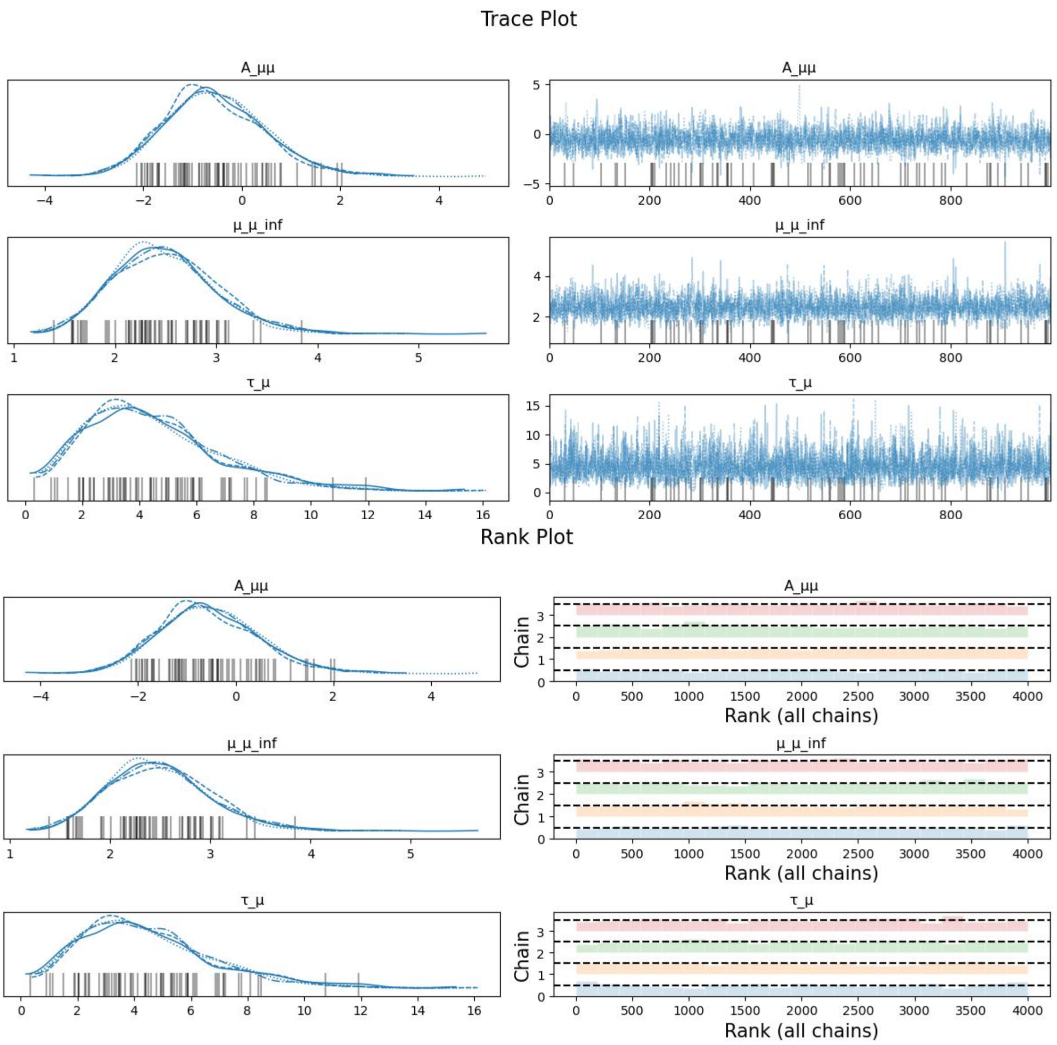

Supplementary Figure 5: Trace Plots and Rank Plots of A_{μ}, $\mu_{\mu,\infty}$, τ_{μ} of fish 9 in the rotation stage. The left column trace plots show the values of each parameter (A_ μ μ , μ _ μ _inf, τ _ μ) over the course of the MCMC iterations. The plots appear to show good mixing, as the chains explore the parameter space without getting stuck in particular regions. The right column trace plots show same parameters but focus on the variation over iterations. The right column rank plots show the rank of each iteration's value within the entire chain for each parameter. The plots show a uniform distribution of ranks across all chains (represented by different colors), which is ideal.

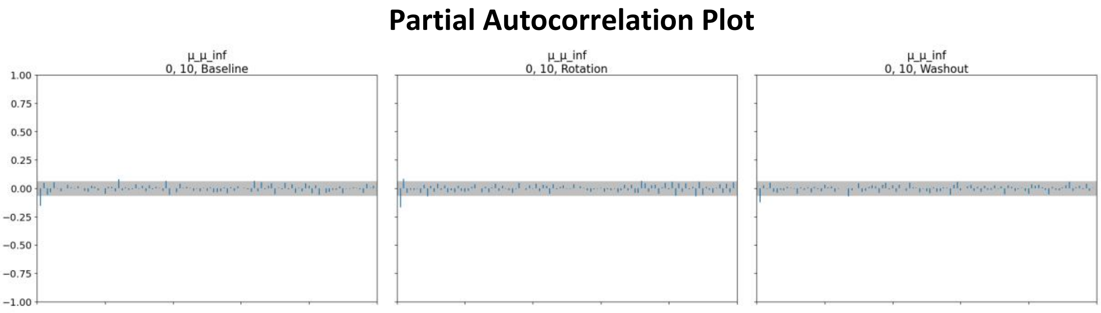

Supplementary Figure 6: Partial Autocorrelation Plot of $\mu_{\mu,\infty}$ of fish 10 in the rotation stage. High autocorrelation in MCMC chains may indicate poor mixing or slow convergence.

5. Posterior distribution

The posterior distribution is the probability distribution of the parameters given the observed data. It combines our prior beliefs about the parameters (prior distribution) with the information from the data (likelihood) to form an updated belief. The posterior distribution represents uncertainty about parameters after observing data.It provides a full probability distribution, not just point estimates. And this allows for probabilistic statements about parameters.

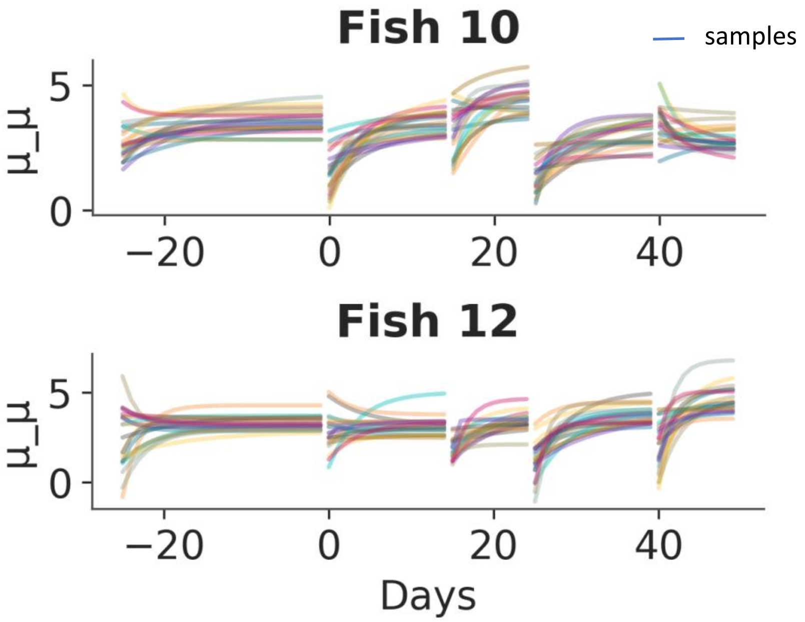

Supplementary Figure 7 Posterior Predictive Checks. This figure displays posterior predictive plots for two fish (Fish 10 and Fish 12) after model fitting with data. Each plot shows 20 different samples drawn from the posterior distribution of the model parameters. Each plot shows 20 different random samples of simulated counts over time, with each color representing a distinct sample. The x-axis represents sessions (based on data), while the y-axis (μ _ μ) represents the model's predicted success/session.

6. The Bayesian model of angular error

6. 1 Priors rationale

Hyperpriors for the **Location Parameter ($\hat{\mu}$)**: $\hat{\mu}$ is the central value or the mean around which the Student's t distribution is centered. It is analogous to the mean in a normal distribution. The location parameter ($\hat{\mu}$) of the Student's t distribution is central to the model, as it governs the mean. $\hat{\mu}$ is defined deterministically by the exponential model fit and thus we did not set priors directly on $\hat{\mu}$ but rather we set hyperpriors on the parameters that govern $\hat{\mu}$. The exponential fit has three hyperparameters: the amplitude (or the amount of change between the first day and the learning asymptote), the learning asymptote, and the time constant.

Hyperpriors for the **Scale Parameter ($\hat{\sigma}$)**: $\hat{\sigma}$ controls the spread or dispersion of the Student's t distribution. It is similar to the standard deviation in a normal distribution. $\hat{\sigma}$ is defined deterministically by the exponential model fit and thus we did not set priors directly on $\hat{\sigma}$ but rather we set hyperpriors on the parameters that govern $\hat{\sigma}$. The exponential fit has three hyperparameters: the amplitude (or the amount of change between the first day and the variance asymptote), the variance asymptote, and the time constant.

Hyperpriors for the **Degrees of Freedom** (ν): ν controls the heaviness of the tails in the distribution. Lower values of ν result in heavier tails (more outliers), while larger values approach the normal distribution. Where ν is fixed across the data and has an exponential prior (0.1).

Amplitude (A_μ), Time Constant (τ_μ): The rationale for choosing these parameters is similar to the previous model. For A_μ , we chose 5.52 (mean of the performance across fish on the last session across stages minus the first session) as the mean and 25.55 (standard deviation of the performance across fish on the last session across stages minus the first session) as the standard deviation as the prior of the normal distributions of each fish in each stage. For τ_μ , we chose 5.41 as the mode and 5.94 as the standard deviation.

Asymptote ($\mu_{\mu,\infty}$): Similarly, the constant term $\mu_{\mu,\infty}$ represents the asymptotic value that performance reaches after many sessions. The difference is that angle is signed value, we opted for a Normal distribution. In the current version we used -6.43 (mean of the performance in the last session across fish and across stage as the mean of the Normal distribution of each fish in each stage) as mean and 14.15 (standard deviation of the performance across fish on the last session across stage) as the standard deviation as the prior of the Normal distribution.

Amplitude of deviation (A_σ):

We chose a Gamma distribution for the prior of the amplitude of deviation (A_σ). The amplitude reflects the difference between the deviation of the performance at the first and last sessions of each stage for each fish. This difference of the deviation is expected to be positive, hence the Gamma distribution. And then we calculated the mode and standard deviation from the real data. We used the standard deviation (taken across fish and across stages) of the performance on the last session minus the performance on the first session as the as mode and standard deviation of the Gamma distribution of each fish in each stage. Ultimately, we chose 25.55 (standard deviation of the performance across fish on the last session across stages minus the first session) as the mode and standard deviation as the prior of the Gamma distributions of each fish in each stage.

Asymptote of deviation ($\sigma_{\mu,\infty}$):

We chose a Gamma distribution for the prior of the asymptote of deviation ($\sigma_{\mu,\infty}$). The asymptote represents the asymptotic value that the deviation of performance reaches after many sessions. This asymptotic value of the deviation is expected to be positive, hence the Gamma distribution. The prior for $\sigma_{\mu,\infty}$ is informed by the standard deviation of performance at the last session of each stage for each fish. We used the standard deviation (taken across fish and

across stages) of the performance on the last session minus the performance on the first session as the as mode and standard deviation of the Gamma distribution of each fish in each stage. Ultimately, we chose 14.15 (standard deviation of the performance across fish on the last session across stages minus the first session) as the mode and standard deviation as the prior of the Gamma distributions of each fish in each stage.

These priors are reasonable because they reflect the constraints and expectations of the model. The normal priors for the amplitude A_{μ} and as the asymptote $\mu_{\mu,\infty}$ allow for variability around a central value (reflecting the variation in initial improvement and asymptotic performance across fish). The Gamma priors for $A_{\sigma,}$ $\sigma_{\mu,\infty}$ and τ_{μ} ensure that these parameters remain positive, as the amplitude of the deviation, the asymptote of the deviation and time constant must be non-negative quantities.

6.2 Prior predictive check

In this case, the angular error of each trial is due to the 45° perturbation added to the control map. Therefore, the reasonable mean value of the simulated data should be broader than -45° to 45°, and the standard deviation should be wider than 90° (in the proper direction) (Supplementary Figure 4).

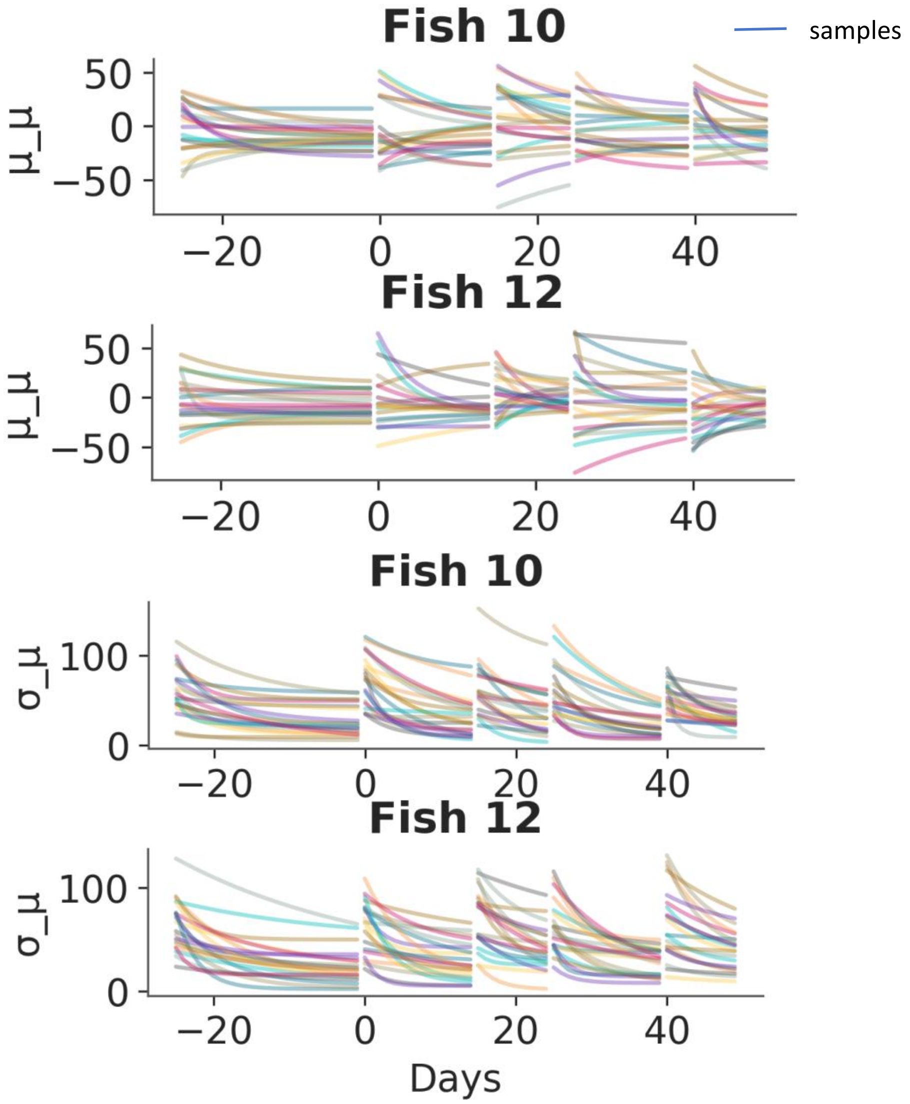

Supplementary Figure 4: Prior Predictive Checks of Angular Error Model for Two Fish. This figure displays prior predictive plots for two randomly selected fish (Fish 10 and Fish 12). Each plot shows 20 different random samples of simulated counts over time, with each color representing a distinct sample. The x-axis represents sessions (from -25 to 50), while the y-axis (μ_μ) represents the model's predicted angular error , the y-axis (σ_μ) represents the model's predicted angular error deviation. in this case, the angular error of each trial is due to the 45° perturbation added to the control map. So the reasonable mean value of the simulated data should be about wider than -45 ~ 45, the standard deviation value of the simulated should be about wider than 90 (proper direction).

6.3 Sampling

	mean	sd	hdi_3%	hdi_97%	mcse_mean	mcse_sd	ess_bulk	ess_tail	r_hat
A_μμ	17.012	8.442	2.067	33.785	0.159	0.126	2849.0	2073.0	1.0
μ_μ_inf	-6.804	5.827	-18.424	2.700	0.129	0.099	2197.0	2076.0	1.0
τ_μ	10.038	5.644	0.923	20.027	0.114	0.080	2300.0	2700.0	1.0

Supplementary Table 1 : Legend: Rows: ‘A_ μ μ’: Amplitude parameter. ‘ μ _ μ _inf’: Asymptote parameter. ‘ τ _ μ’: Time constant parameter. Columns: ‘mean’: Posterior mean. ‘sd’: Posterior standard deviation. ‘hdi_3%’: 3% highest density interval. ‘hdi_97%’: 97% highest density interval. ‘mcse_mean’: Monte Carlo standard error of the mean. ’ mcse_sd’: Monte Carlo standard error of the standard deviation. ‘ess_bulk’: Effective sample size (bulk). ‘ess_tail’: Effective sample size (tail). r_hat: R-hat convergence statistic.

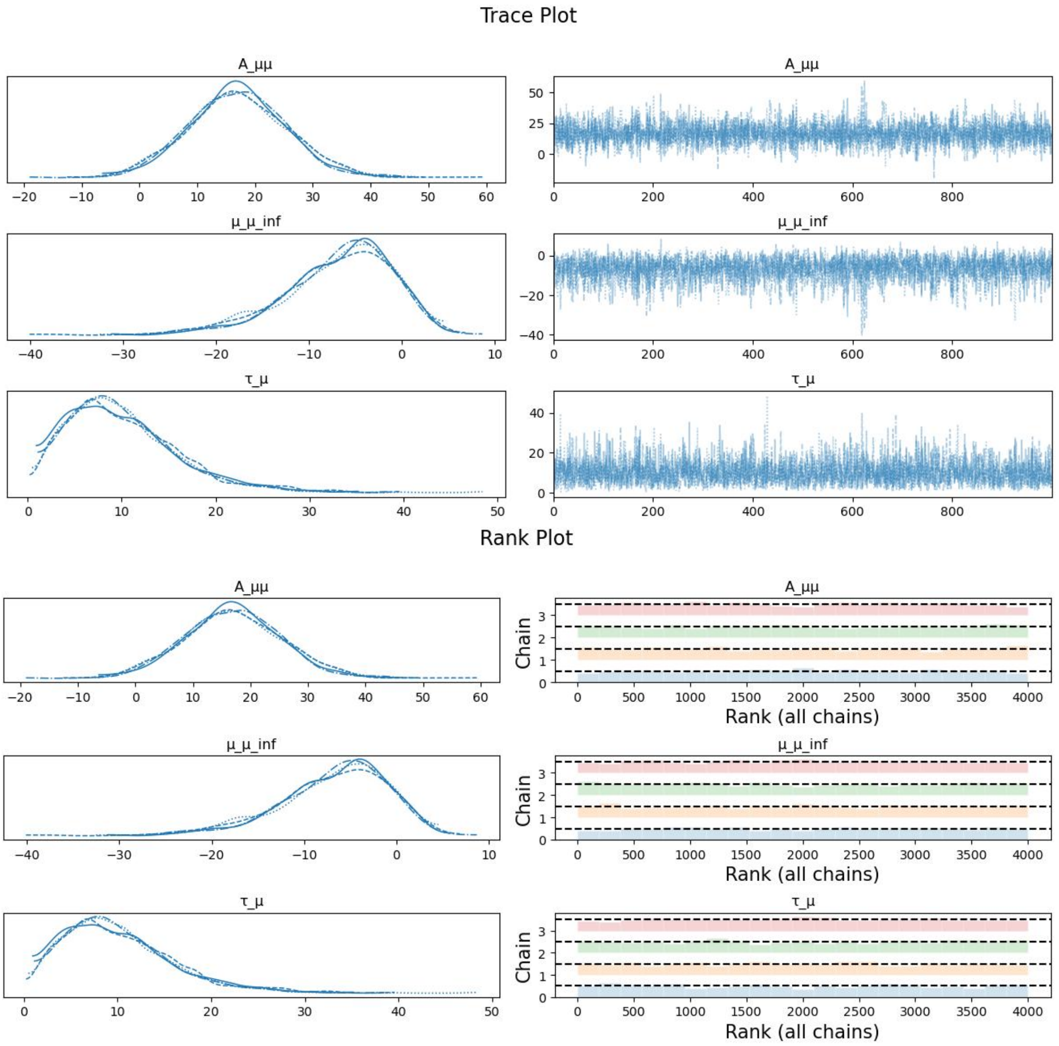

Supplementary Figure 5: Trace Plots and Rank Plots of A_{μ}, $\mu_{\mu,\infty}$, τ_{μ} of fish 9 in the rotation stage. The left column trace plots show the values of each parameter (A_ μ μ, μ _ μ _inf, τ _ μ) over the course of the MCMC iterations. The plots appear to show good mixing, as the chains explore the parameter space without getting stuck in particular regions. The right column trace plots show same parameters but focus on the variation over iterations. The right column rank plots show the rank of each iteration's value within the entire chain for each parameter.

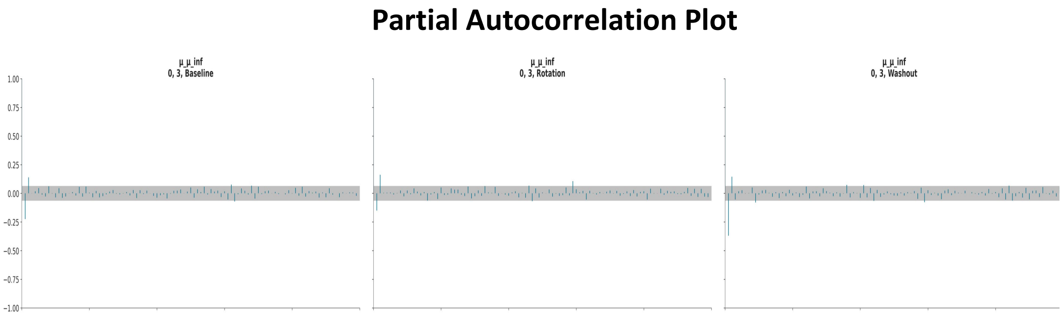

Supplementary Figure 6: Partial Autocorrelation Plot of $\mu_{\mu,\infty}$ of fish 10 in the rotation stage. High autocorrelation in MCMC chains may indicate poor mixing or slow convergence.

6.4 Posterior distribution

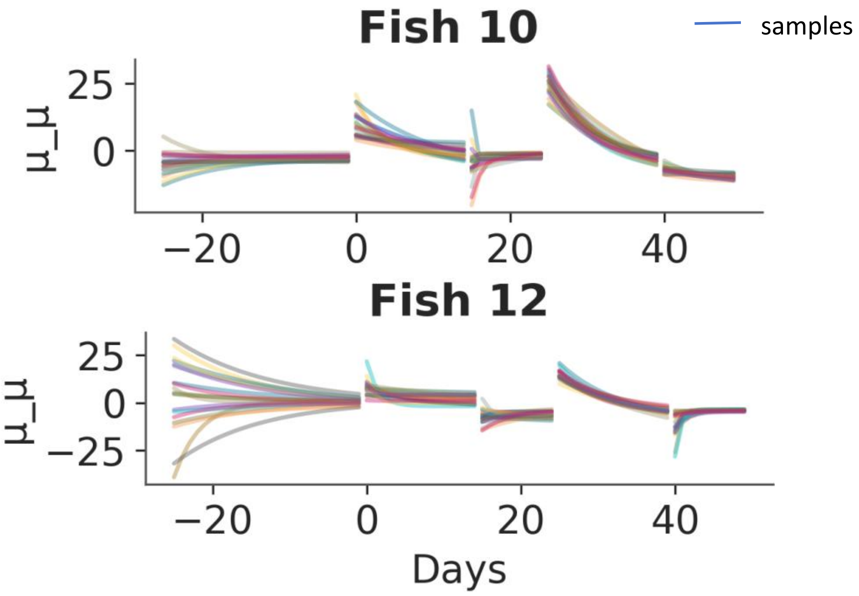

Supplementary Figure 7 Posterior Predictive Checks. This figure displays posterior predictive plots for two fish (Fish 10 and Fish 12) after model fitting with data. Each plot shows 20 different samples drawn from the posterior distribution of the model parameters. Each plot shows 20 different random samples of simulated counts over time, with each color representing a distinct sample. The x-axis represents sessions (based on data), while the y-axis (μ _ μ) represents the model's predicted median angular error of one session.

7. The Bayesian model of distance travelled

7.1 Priors rationale

Hyperpriors for the **Shape Parameter ($\hat{\mu}$)**: $\hat{\mu}$ controls the shape of the gamma distribution. A higher shape value results in a more right-skewed distribution. $\hat{\mu}$ is defined deterministically by the exponential model fit and thus we did not set priors directly on $\hat{\mu}$ but rather we set hyperpriors on the parameters that govern $\hat{\mu}$. The exponential fit has three hyperparameters:

the amplitude (or the amount of change between the first day and the learning asymptote), the learning asymptote, and the time constant.

Hyperpriors for the **Rate Parameter ($\hat{\sigma}$)**: $\hat{\sigma}$ controls the rate of decay or the scale of the distribution. It is the inverse of the scale parameter, so a higher rate leads to a faster decay (narrower distribution). $\hat{\sigma}$ is defined deterministically by the exponential model fit and thus we did not set priors directly on $\hat{\sigma}$ but rather we set hyperpriors on the parameters that govern $\hat{\sigma}$. The exponential fit has three hyperparameters: the amplitude (or the amount of change between the first day and the variance asymptote), the variance asymptote, and the time constant.

Time Constant (τ_{μ}), Asymptote ($\mu_{\mu,\infty}$), Amplitude of deviation (A_{σ}), Asymptote of deviation ($\sigma_{\mu,\infty}$) : The rationale for choosing Gamma distributions for the prior of these parameters is similar to the previous models. For τ_{μ}, we chose 7.56 as the mode and 8.20 as the standard deviation. For $\mu_{\mu,\infty}$, we chose 4.70 as the mode and 2.24 as the standard deviation. For A_{σ}, we chose 3.47 as the mode and standard deviation. For $\sigma_{\mu,\infty}$, we chose 2.24 as the mode and standard deviation.

Amplitude (A_{μ}):

We chose a left-censored normal (l-cens) distribution for the prior of the amplitude (A_{μ}). The left-censored normal (L-cens) distribution is a variation of the normal (Gaussian) distribution that accounts for left-censoring, meaning that values below a certain threshold are not observed or are recorded as that threshold value. This type of distribution is commonly used when there's a lower bound on the data, such as in scenarios where measurements cannot fall below a certain detection limit (in this case, distance travelled cannot measure values below the optimal distance). The amplitude reflects the difference between performance at the first and last sessions of each stage for each fish. We used the mean of the performance across fish on the last session across stages minus the first session as the location parameter of the l-cens normal distributions of each fish in each stage. We used the standard deviation (taken across fish and across stages) of the performance on the last session minus the performance on the first session as the scale parameter of the l-cens. normal distribution of each fish in each stage. And we used -1 * the asymptote of the learning performance as the censoring point (The threshold below which data is censored). Ultimately, we chose 2.40 as the mode, 3.47 as the standard deviation and 1 * the asymptote of the learning performance of the sample as the prior of the l-cens normal distributions of each fish in each stage. Since pymc parameterizes Truncted Normal distribution using shape, rate (Bayesian regression with truncated or censored data — PyMC example gallery) and censoring point, we converted our chosen mode and standard deviation into shape and rate using standard formulas for this reparatemerization.

7.2 Prior predictive check

In this case, the distance traveled in each trial is based on the total trajectory length of the FOV. Therefore, the reasonable mean value of the simulated data should be broader than 2.5 m (the optimal distance) to a finite positive value (the theoretical maximum distance the FOV can travel in three minutes), with the standard deviation also being broader than the finite positive value

(Supplementary Figure 5).

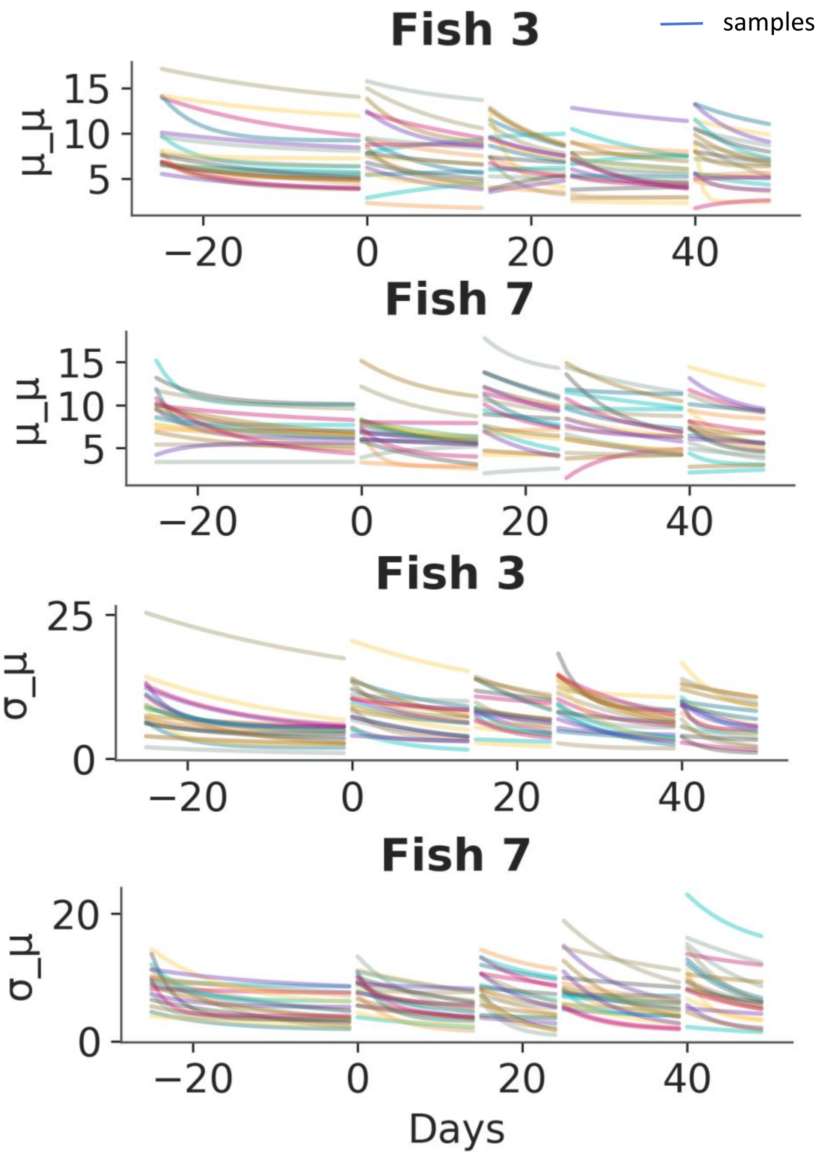

Supplementary Figure 4: Prior Predictive Checks of Distance Travelled Model for Two Fish. This figure displays prior predictive plots for two randomly selected fish (Fish 3 and Fish 7). Each plot shows 20 different random samples of simulated counts over time, with each color representing a distinct sample. The x-axis represents sessions (from -25 to 50), while the y-axis (μ_μ) represents the model's predicted distance travelled, the y-axis (σ_μ) represents the model's predicted distance travelled deviation. in this case, the distance travelled of each trial is based on the total trajectory length of the FOV. So the reasonable mean value of the simulated data should be about wider than 2.5 (optimal distance) ~ the finite positive value (theoretical maximum distance of FOV travel of across fish in three minutes), the standard deviation value of the simulated should be about wider than the finite positive value.

7.3 Sampling

	mean	sd	hdi_3%	hdi_97%	mcse_mean	mcse_sd	ess_bulk	ess_tail	r_hat
A_μμ	2.754	1.675	-0.395	5.813	0.020	0.016	7223.0	2781.0	1.0
μ_μ_inf	4.023	1.015	2.105	5.956	0.012	0.009	6775.0	2740.0	1.0
τ_μ	15.199	7.282	3.468	28.378	0.098	0.076	5459.0	3331.0	1.0

Supplementary Table 1 : Legend: Rows: 'A_μμ': Amplitude parameter. 'μ_μ_inf': Asymptote parameter. 'τ_μ': Time constant parameter. Columns: 'mean': Posterior mean. 'sd': Posterior standard deviation. 'hdi_3%': 3% highest density interval. 'hdi_97%': 97% highest density interval. 'mcse_mean': Monte Carlo standard error of the mean. ' mcse_sd': Monte Carlo standard error of the standard deviation. 'ess_bulk': Effective sample size (bulk). 'ess_tail': Effective sample size (tail). r_hat: R-hat convergence statistic.

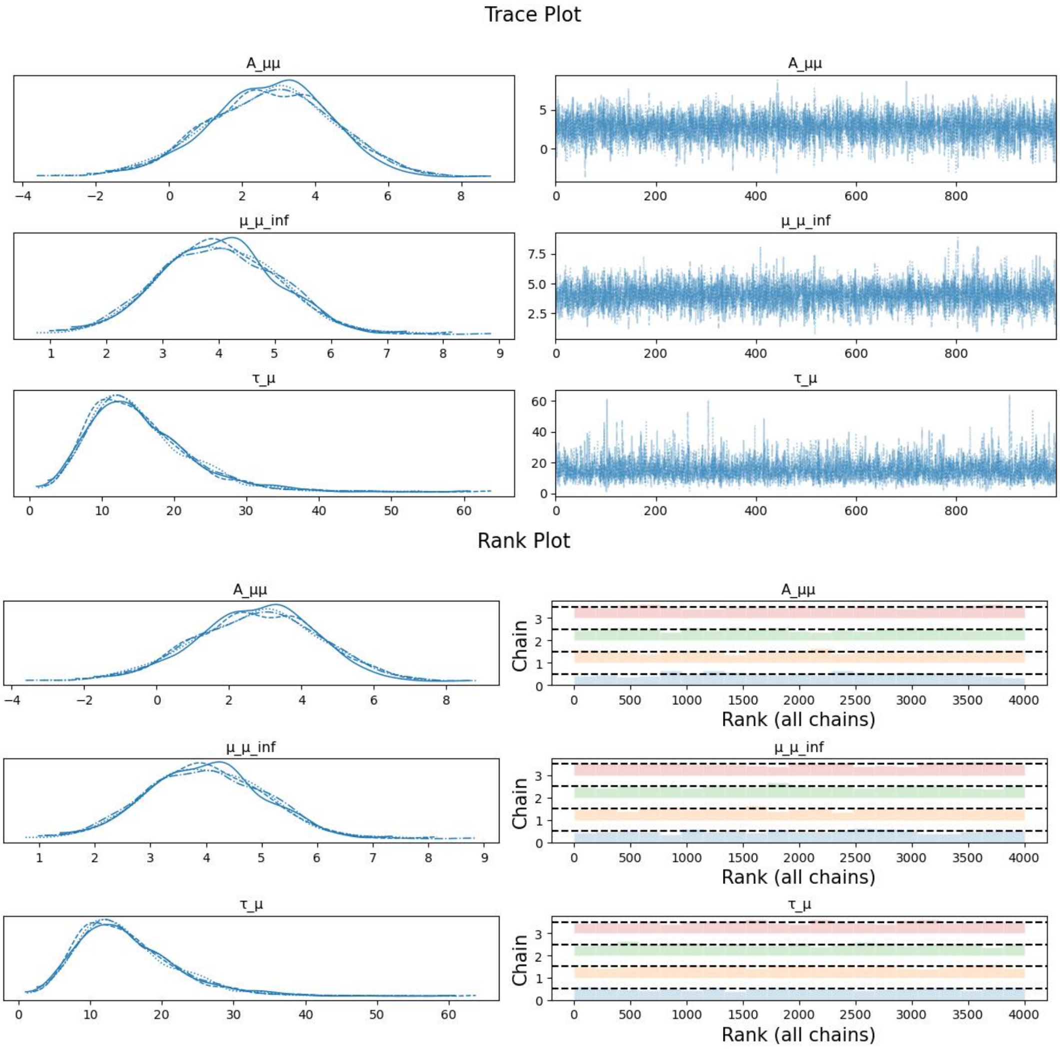

Supplementary Figure 5: Trace Plots and Rank Plots of A_{μ}, $\mu_{\mu,\infty}$, τ_{μ} of fish 9 in the rotation stage. The left column trace plots show the values of each parameter (A_μμ, μ_μ_inf, τ_μ) over the course of the MCMC iterations. The plots appear to show good mixing, as the chains explore the parameter space without getting stuck in particular regions. The right column trace plots show same parameters but focus on the variation over iterations. The right column rank plots show the rank of each iteration's value within the entire chain for each parameter.

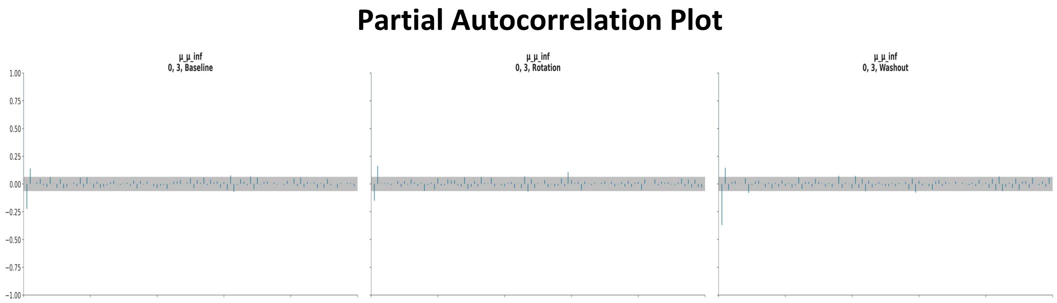

Supplementary Figure 6: Partial Autocorrelation Plot of $\mu_{\mu,\infty}$ of fish 10 in the rotation stage. High autocorrelation in MCMC chains may indicate poor mixing or slow convergence.

7.4 Posterior distribution

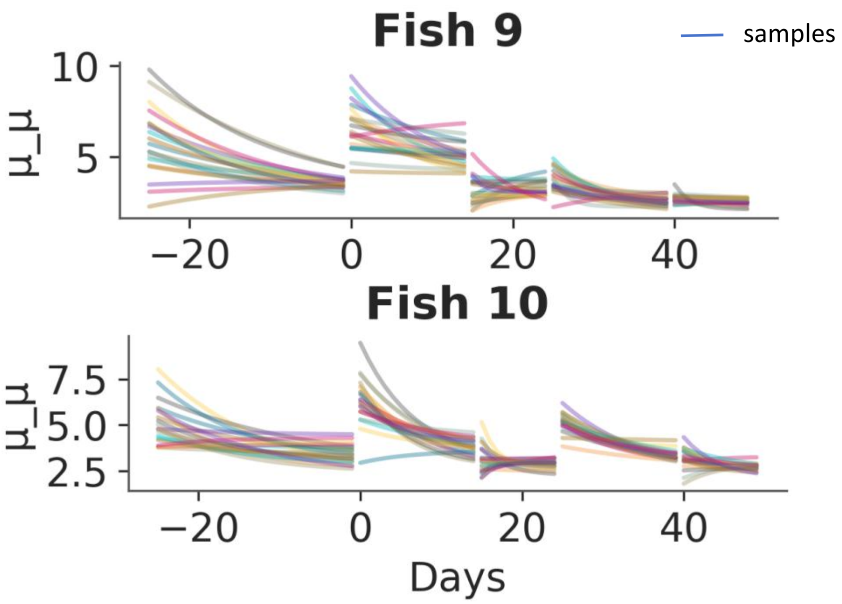

Supplementary Figure 7 Posterior Predictive Checks. This figure displays posterior predictive plots for two fish (Fish 10 and Fish 12) after model fitting with data. Each plot shows 20 different samples drawn from the posterior distribution of the model parameters. Each plot shows 20 different random samples of simulated counts over time, with each color representing a distinct sample. The x-axis represents sessions (based on data), while the y-axis (μ _ μ) represents the model's predicted median distance travelled of one session.

8. Fish can adapt to -45° Rotation

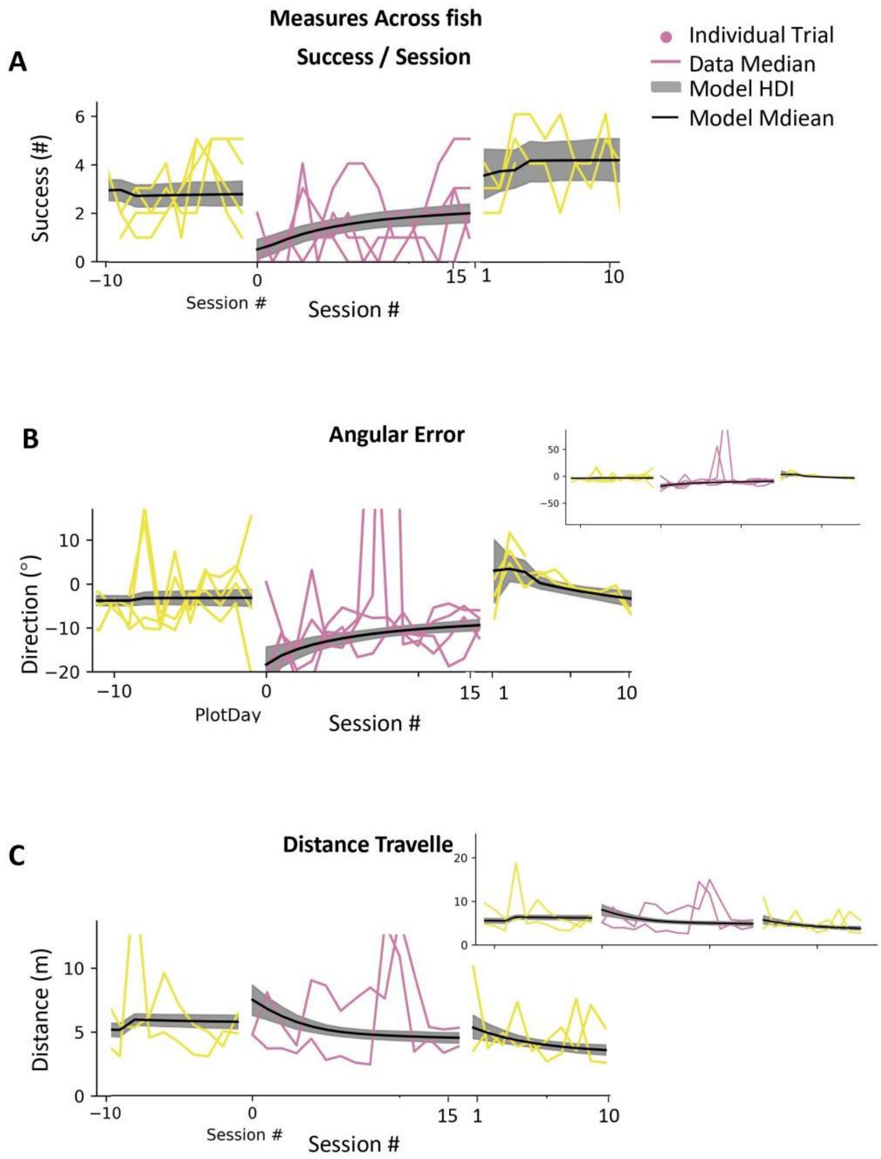

Supplementary Figure 8 Goldfish can adapt to -45 rotation. A-C. The colorful lines are the raw median data in one session of the performance of across fish. The black lines are the model estimations of the median performance across fish. The grey patches are HDI of the model estimation of the median performance across fish.

9. Example videos of FOV adaptation experiments

https://doi.org/10.6084/m9.figshare.27080731.v1

[Example videos of FOV adpatation (figshare.com)]

10. Reference

Gelman A, Vehtari A, Simpson D, Margossian CC, Carpenter B, Yao Y, Kennedy L, Gabry J, Bürkner P-C, Modrák M. Bayesian Workflow. arXiv2020.

Maresch J, Mudrik L, Donchin O. Measures of explicit and implicit in motor learning: what we know and what we don't. *Neuroscience & Biobehavioral Reviews* 128: 558–568, 2021.

Martin OA, Kumar R, Lao J. Bayesian Modeling and Computation in Python. 1st ed. Chapman and Hall/CRC.
